# Fractional epidemic model of coronavirus disease with vaccination and crowding effects

**DOI:** 10.1038/s41598-024-58192-7

**Published:** 2024-04-08

**Authors:** Suhail Saleem, Muhammad Rafiq, Nauman Ahmed, Muhammad Shoaib Arif, Ali Raza, Zafar Iqbal, Shafiullah Niazai, Ilyas Khan

**Affiliations:** 1 Department of Mathematics, Air University, PAF Complex E-9, Islamabad, 44000 Pakistan; 2 Department of Mathematics, Faculty of Science and Technology, University of Central Punjab, Lahore, Pakistan; 3 Department of Mathematics and Statistics, The University of Lahore, Lahore, Pakistan; 4 Department of Mathematics, University of Chanab, Gujrat, Pakistan; 5 Department of Mathematics, Education Faculty, Laghman University, Mehtarlam City, 2701 Laghman Afghanistan; 6 Department of Mathematics, College of Science Al-Zulfi Majmaah University, 11952 Al-Majmaah, Saudi Arabia; 7 Department of Computer Science and Mathematics, Lebanese American University, Beirut, 1102-2801 Lebanon; 8 Department of Mathematics, Mathematics Research Center, Near East University, Near East Boulevard, 99138 Nicosia/Mersin 10, Turkey; 9 Department of Mathematics, Saveetha School of Engineering, SIMATS, Chennai, Tamil Nadu India

**Keywords:** SIVR model, Infection dynamics, Precautionary measures, Non-linear differential equation, Existence and uniqueness, Basic reproductive number, Stability, Non-standard finite difference approach, Biological models, Software, Mathematics and computing

## Abstract

Most of the countries in the world are affected by the coronavirus epidemic that put people in danger, with many infected cases and deaths. The crowding factor plays a significant role in the transmission of coronavirus disease. On the other hand, the vaccines of the covid-19 played a decisive role in the control of coronavirus infection. In this paper, a fractional order epidemic model (SIVR) of coronavirus disease is proposed by considering the effects of crowding and vaccination because the transmission of this infection is highly influenced by these two factors. The nonlinear incidence rate with the inclusion of these effects is a better approach to understand and analyse the dynamics of the model. The positivity and boundedness of the fractional order model is ensured by applying some standard results of Mittag Leffler function and Laplace transformation. The equilibrium points are described analytically. The existence and uniqueness of the non-integer order model is also confirmed by using results of the fixed-point theory. Stability analysis is carried out for the system at both the steady states by using Jacobian matrix theory, Routh–Hurwitz criterion and Volterra-type Lyapunov functions. Basic reproductive number is calculated by using next generation matrix. It is verified that disease-free equilibrium is locally asymptotically stable if ${R}_{0}<1$ and endemic equilibrium is locally asymptotically stable if ${R}_{0}>1$. Moreover, the disease-free equilibrium is globally asymptotically stable if ${R}_{0}<1$ and endemic equilibrium is globally asymptotically stable if ${R}_{0}>1$. The non-standard finite difference (NSFD) scheme is developed to approximate the solutions of the system. The simulated graphs are presented to show the key features of the NSFD approach. It is proved that non-standard finite difference approach preserves the positivity and boundedness properties of model. The simulated graphs show that the implementation of control strategies reduced the infected population and increase the recovered population. The impact of fractional order parameter $\alpha$ is described by the graphical templates. The future trends of the virus transmission are predicted under some control measures. The current work will be a value addition in the literature. The article is closed by some useful concluding remarks.

## Introduction

The severe acute respiratory syndrome (SARS) was initially detected in Asia in 2003. Then, this infection spread to the other parts of the world. Main symptoms of the SARS virus contain high body temperature, dysentery, discomfort, dry cough and respiratory problems etc.^1^. It is an air-borne disease, that spreads by the tiny droplets produced by coughing, sneezing, laughing or talking infected person. These symptoms vary from mild to severe depending upon the immunity, health condition and phase of the virus. The SARS cov2 or Covid-19 was started in December 2019 from Wuhan, China. This virus was similar to that of the SARS virus. It is considered that Covid-19 was spread in humans via the bats. The initial symptoms of this virus was similar to that of the pneumonia virus^2,3^. This virus propagated to the other parts of the world, eventually it reached in the United States on January 20, 2020. It was declared as pandemic on March 11, 2020 by the WHO. In the beginning, the value of ${R}_{0}$ was $2.5,$ apporoximately. Because of its crown like structure, it is called as Corona virus. The spike-protein attacks on the cells of the respiratory system. Gradually, Covid-19 introduces its contaminated RNA into the human bodies. Then the virus replicates and multiplies its production in a short span of time. Everyone is susceptible to the Corona virus, but the aged and sick people are the easy target of this disease. The people with low immunity and respiratory issues, may acquire it fastly. This disease may reoccur in an individual which increases its rate of fatality. The USA acquired the highest rate of infection in the world, while Brazil was recorded with the highest mortality rate and Vietnam with the lowest in the world. Covid-19 is a different virus than influenza-A or Influenza-B viruses. The strain of it is similar to SARS Cov2 and its signs and symptoms appear with in 2 to 14 days after the vulnerability. It has been observed that, COVID-19 is more infectious than the other viruses of the family. One may lose sense of taste or smell after getting the virus. Some other issues like fever and respiratory problems may also develop in the patients. So, the importance of preventive measures for these types of diseases has been increased. Social distancing, wearing face masks and hand sanitizing can control the disease dynamics, considerably^4^. Vaccination imparts an important role in controlling the disease. But it is not sure that a vaccinated person cannot receive the infection. It is investigated that the people with strong immunity can restrict the propagation of the disease^5^. Ahmed et al. considered the SEQIIR model in 2021 to study the dynamics of the corona virus. They used ODEs and FDEs models for the deeper insight of the disease phenomena^6^. Likewise, In 2021, Hassan et al. suggested the SIIR compartmental model to examine the waves of the disease in Texas, USA^7^. Alqarni et al. described the DSIARB epidemic model to notice the dynamics of Covid-19 in the Kingdom of Saudi Arabia^8^. Similarly, in Brazil Savi and his co-authors studied an SEIRDC epidemic model to check the propagation of the virus^9^. Tiwari et al. explored the effect of quarantine on the dynamics of the disease in India. They tested the disease dispersion by analyzing the SEIRD model^10^. Warbhe et al. in 2021, inspected a SIRM model to study the economic losses due to Covid-19^11^. In 2021, Daniel studied the compartmental (SEIQCRW) model to explore the dynamics of the infection Covid-19 with diffusion process^12^. Prathumwan et al. considered a SLIQHR compartmental model to examine the hazards of the corona virus by adopting the safety measures^13^. The Covid-19 influenced the world economy badly. Balike explored the economic effects of the virus in the model in Congo by studying the SEIHQR model^14^. Likewise, in Egypt, Raslan discussed SEHQIR compartmental model to explore the virus propagation of the new invariant of the COVID-19^15^. Chen et al. proposed a new compartment model (BHRP) to simulate the disease dynamics^16^. In Indonesia, Sinaga et al. contemplated a model (SEIR) for examining the structural properties of the coronavirus^17^. James et al. highlighted the pros and cons of the mathematical modeling in designing the health policies^18^. Ameen et al. proposed a fractional order mathematical model (SSLLIPD) for investigating the communication of the virus^19^. Similarly, different compartmental models were suggested to verify the propagation of Covid-19 in the society^20,21^. Kahn et al. studied the model disease dynamics by including the vaccination strategy in the model^22^. Nave and co-authors designed a model to study the stability of the virus by adopting the fast-slow classification strategy^23^. Kim et al. explored that social distancing, isolation and early case detection plays a vital key role in controlling the disease dynamics of Covid-19^24^. Das studied the stability analysis of fractional order Covid-19 model^25^. Machado et al. studied the rare and extreme events during the pandemic of the Covid-19^26^. On the same lines, many researchers proposed different types of models with the consideration of various important parameters to examine the spread of the virus^27^. Quaranta and co-authors analyzed various multi-scale territorial models for the deeper understanding of the virus propagation^28^. Many research studies conducted to investigate the propagation of corona-like diseases^29–33^. Different epidemic models and strategies are designed to illustrate the transmission dynamics of the Covid-19^34–37^.

Mathematical models don't offer a definitive cure for infectious diseases, they only serve to simulate various scenarios and dynamics, resilience assessment, control and effective detection strategies. Timely and appropriate interventions are crucial for mitigating the social impact of diseases by controlling their spread. Many mathematical models in literature have been proposed to understand and predict the spread of infectious diseases, with the objective to flatten the infection curve and reduce mortality rates.

Fractional calculus, a field extending classical derivatives and integrals to fractional orders, has a rich history dating back to Leibniz's inquiries in 1695. This branch of applied mathematics deals with real-world phenomena modeled by non-integer-order derivatives and increasing attention globally. Various fractional derivatives, with or without singular kernels, have been developed and extensively employed to model diverse real-life problems. These derivatives include the Caputo, Riemann–Liouville, Katugampola, Caputo–Fabrizio, and Atangana–Baleanu derivatives. The applications of fractional calculus may be seen in^50–54^.

In^38^, Ali and co-authors presented SIVR (Susceptible, Infected, Vaccinated, Recovered) epidemic ODE model for analyzing and predicting the COVID-19 but as we know that the fractional order derivative is much more accurate, factual and empirical when compared with the integer order cases.

Researchers have utilized fractional calculus to enhance epidemic models, incorporating memory effects and studied the disease dynamics more accurately. The efficacy of fractional operators has led to their widespread application across various disciplines such as Finance, Engineering, Biology, and Medicine. In the context of COVID-19, where uncertainties abound, fractional calculus offers advantages over integer-order derivatives. Fractional operators, characterized by their non-local nature, are better suited to capturing complex and unpredictable systems. The memory and hereditary properties inherent in fractional order models enable more realistic representations of infectious disease dynamics, incorporating past information for better predictions. Recent developments in fractional calculus may be observed in^55–59^.

To fill these gaps and to address the challenges arise in the COVID-19 modeling, the present study focuses on analyzing an SIVR epidemic model using the Caputo fractional order operator. This choice is justified because the Caputo derivative has the ability to accommodate local initial conditions and it is compatible with biological and physical principles.

The primary objective of this research is to assess the impact of vaccination and crowding effect on COVID-19 dynamics in the perspective of fractional calculus. The insights gained from this study could assist in strategic planning by governments and public health authorities to bridge immunization gaps and prevent future outbreaks. Furthermore, this fractional order model will contribute to the ongoing research in COVID-19 mathematical modeling and will also assist the researchers to stimulate interest in fractional calculus modeling and mathematical epidemiology.

The design of our paper is as follows. In “Description of the model” section, we have discussed the mathematical model and performed its analysis. Then, in the sub-sections, positivity, boundedness, existence, and uniqueness are studied. In “Qualitative analysis of the proposed model” section, qualitative analysis of the proposed model (local and global stability of the model) is presented. In “Numerical simulations” section, numerical simulations are presented to analyze the dynamics of the virus, graphically. In “Numerical method” section, numerical method (Grunwald–Letnikov non-standard finite difference method) of the proposed model is presented. Then, in the sub-sections, positivity and boundedness of the numerical method are discussed. Finally, the concluding remarks are presented in the closing section. Now, we quote some basic definitions of fractional calculus.

### Definition 1

(Caputo Fractional Derivative) The Caputo derivative of fractional order $\alpha$ of function $f(t)$ is defined as.

$${}_{a}{}^{c}{D}_{t}^{\alpha }f\left(t\right)=\frac{1}{\Gamma (n-\alpha )}{\int }_{a}^{t}\frac{{f}^{\left(n\right)}(x)}{{\left(t-x\right)}^{\alpha +1-n}}dx,\text{ where }n-1<\alpha <n\in {\mathbb{N}}.$$

### Laplace transform of Caputo fractional derivative

The Laplace transformation of Caputo fractional differential operator of order $\alpha$ is given by:

$$L\left\{{}_{0}{}^{c}{D}_{t}^{\alpha }f\left(t\right)\right\}={s}^{\alpha }F\left(s\right)-\sum_{k=0}^{n-1}{s}^{k-n-1}{f}^{\left(k\right)}\left(0\right),\text{ where }n-1<\alpha <n\in {\mathbb{N}}.$$

#### Definition 2

(The Mittag–Leffler Functions) Two parametric Mittag–Leffler function is represented by the series.

$${E}_{\alpha ,\beta }\left(z\right)=\sum_{k=0}^{\infty }\frac{{z}^{k}}{\Gamma (\mathrm{\alpha k}+\upbeta )} , \alpha ,\beta >0 , \alpha ,\beta \in {\mathbb{R}},z\in {\mathbb{C}}.$$

### Laplace transformation of the Mittag–Leffler Functions

Laplace transformation of the Mittag–Leffler function is given by.

$$L\left\{{t}^{\beta -1}{E}_{\alpha ,\beta }\left(M{t}^{\alpha }\right)\right\}=\frac{{s}^{\alpha -\beta }}{{s}^{\alpha }-M}.$$

For further definitions and properties of fractional calculus, see^39–45^.

## Description of the model

In this segment, we present the fractional epidemic system of coronavirus disease.

**Figure 1:**
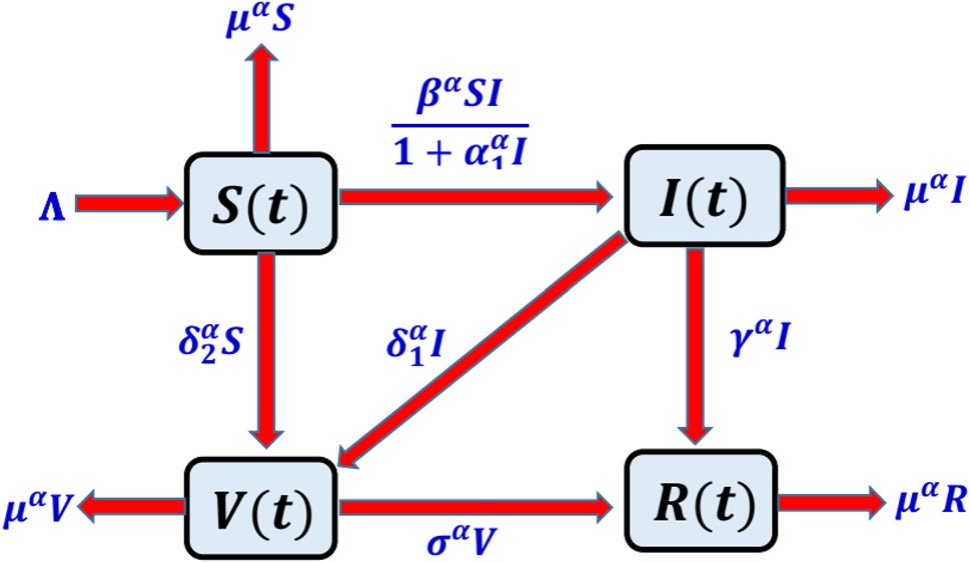
Flow map of coronavirus model.

In the proposed model, the whole human population $N(t)$ is classified into four subclasses as $S(t)$ (Susceptible class), $I(t)$ (Infected class), $V(t)$ (Vaccinated class), and $R(t)$ (Recovered or immune class). The principle of mass action is taken into account for the infection dynamics in the society. The transmission map of the model is shown in Fig. 1.

Parameters of the model are described as follows $\Lambda N$ (the recruitment rate of the population),${\beta }^{\alpha }I$ (the force of infection of virus), $\frac{1}{1+{\alpha }_{1}^{\alpha }I}$ (the crowding effect of population on the virus), ${\mu }^{\alpha }$ (the rate of mortality due to virus or natural of each subpopulation), ${\delta }_{1}^{\alpha }$ (the rate at which infected population got vaccination during the period of quarantine, or isolation, etc.), ${\delta }_{2}^{\alpha }$ (the rate at which susceptible population got vaccination under the program launched by World Health Organization (WHO)), ${\gamma }^{\alpha }$ (the rate at which infected population may recover due to its internal immunity and natural circumstances), and ${\sigma }^{\alpha }$ (the rate of doses in the population who recovered or got immune after vaccination). Deterministic model is based on the following assumptions. Susceptible population becomes immune against the disease after vaccination. Recovered population do not become infected. Only direct contact of infected individuals and susceptible population is considered in this model, all other types of interactions are neglected.

### Model equations

The system of equations obtained from the transmission map of the virus is as follows:

1 $$\begin{aligned} & {}_{0}^{c} D_{t}^{\alpha } S\left( t \right) = {\Lambda } - \frac{{\beta^{\alpha } SI}}{{1 + \alpha_{1}^{\alpha } I }}{-}\left( {\delta_{2}^{\alpha } + \mu^{\alpha } } \right)S\quad {\text{for}}\quad {\text{t}} \ge 0, \\ & {}_{0}^{c} D_{t}^{\alpha } I\left( t \right) = \frac{{\beta^{\alpha } SI}}{{1 + \alpha_{1}^{\alpha } I }} - \left( {\gamma^{\alpha } + \delta_{1}^{\alpha } + \mu^{\alpha } } \right)I\quad {\text{for}}\quad {\text{t}} \ge 0, \\ & {}_{0}^{c} D_{t}^{\alpha } V\left( t \right) = \delta_{2}^{\alpha } S + \delta_{1}^{\alpha } I - \left( {\sigma^{\alpha } + \mu^{\alpha } } \right)V\quad {\text{for}}\quad {\text{t}} \ge 0, \\ & {}_{0}^{c} D_{t}^{\alpha } R\left( t \right) = \gamma^{\alpha } I + \sigma^{\alpha } V - \mu^{\alpha } R\quad {\text{for}}\quad {\text{t}} \ge 0. \\ \end{aligned}$$

### Invariant region

The total dynamics of the system (1) is obtained by adding the four equations as follows

$${}_{0}{}^{c}{D}_{t}^{\alpha }S\left(t\right)+ {}_{0}{}^{c}{D}_{t}^{\alpha }I\left(t\right)+ {}_{0}{}^{c}{D}_{t}^{\alpha }V\left(t\right)+ {}_{0}{}^{c}{D}_{t}^{\alpha }R\left(t\right) =\Lambda -{\mu }^{\alpha }{\text{N}}.$$

where $$N\left(t\right)=S\left(t\right)+I\left(t\right)+V\left(t\right)+R(t)$$.

Finally, we have

$${}_{0}{}^{c}{D}_{t}^{\alpha }N\left(t\right)=\Lambda -{\mu }^{\alpha }{\text{N}}.$$

Hence, $$N\left(t\right)\le M,$$ whenever $$t \to \infty .$$

The feasible region for the system (1) is defined by

$$\Omega =\left\{S\left(t\right),I\left(t\right),V\left(t\right),R\left(t\right)\in {R}_{+}^{4}: N\left(t\right)\le M\right\}.$$

### Properties

In this section, we shall study the basic properties of our proposed model (1). The model will be biological meaningful if all the variables are non-negative for $t\ge 0.$ It means that solution with non-negative initial conditions will remain non-negative for all time. We ensure this result by Theorem 1.

#### Theorem 1

(Positivity) For any initial data $\left(S\left(0\right),I\left(0\right),V\left(0\right),R\left(0\right)\right)\in {\mathbb{R}}_{4}^{+} ,$ the solution $\left(S\left(t\right),I\left(t\right),V\left(t\right),R\left(t\right)\right)$ for the system (1) is positive invariant in ${\mathbb{R}}_{4}^{+}$.

#### Proof

We define the norm as $f_{\infty } = \mathop {\sup }\nolimits_{{t \in D_{f} }} \left| {f\left( t \right)} \right|.$

Firstly, consider the class $S\left(t\right),$

$${}_{0}^{c} D_{t}^{\alpha } S\left( t \right) = {\Lambda } - \frac{{\beta^{\alpha } SI}}{{1 + \alpha_{1}^{\alpha } I }} - \left( {\delta_{2}^{\alpha } + \mu^{\alpha } } \right)S,$$

$${}_{0}^{c} D_{t}^{\alpha } S\left( t \right) \ge - \left( {\delta_{2 }^{\alpha } + \mu^{\alpha } + \frac{{\beta^{\alpha } \left| I \right|}}{{1 + \alpha_{1}^{\alpha } \left| I \right| }}} \right)S,\quad \forall \;{\text{t}} \ge 0,$$

$${}_{0}^{c} D_{t}^{\alpha } S\left( t \right) \ge - \left( {\delta_{2 }^{\alpha } + \mu^{\alpha } + \frac{{\beta^{\alpha } \mathop {\sup }\nolimits_{{t \in D_{\lambda } }} \left| I \right|}}{{1 + \alpha_{1}^{\alpha } \mathop {\sup }\nolimits_{{t \in D_{\lambda } }} \left| I \right| }}} \right)S,\quad \forall {\text{t }} \ge 0,{ }$$

$${}_{0}{}^{c}{D}_{t}^{\alpha }S\left(t\right)\ge -\left({\delta }_{2 }^{\alpha }+{\mu }^{\alpha }+\frac{{\beta }^{\alpha }{I}_{\infty }}{1+{\alpha }_{1}^{\alpha }{I}_{\infty } }\right)S , \forall \mathrm{ t }\ge 0.$$

Let $${M}_{1}={\delta }_{2 }^{\alpha }+{\mu }^{\alpha }+\frac{{\beta }^{\alpha }{I}_{\infty }}{1+{\alpha }_{1}^{\alpha }{I}_{\infty }}$$,

then

$${}_{0}^{c} D_{t}^{\alpha } S\left( t \right) \ge - M_{1} S\left( t \right),$$

$${}_{0}^{c} D_{t}^{\alpha } S\left( t \right) + M_{1} {\text{S}}\left( {\text{t}} \right) \ge 0.$$

By taking Laplace transformation on both sides of the above expression, we get

$$L\{ {}_{0}^{c} D_{t}^{\alpha } S\left( t \right)\} + M_{1} {\text{L}}\left\{ {{\text{S}}\left( {\text{t}} \right)} \right\} \ge 0,$$

$$s^{\alpha } L\left\{ {S\left( t \right)} \right\} - s^{\alpha - 1} S\left( 0 \right) + M_{1} L\left\{ {S\left( t \right)} \right\} \ge 0,$$

$$\begin{aligned} & \left( {s^{\alpha } + M_{1} } \right)L\left\{ {S\left( t \right)} \right\} \ge s^{\alpha - 1} S\left( 0 \right), \\ & L\left\{ {S\left( t \right)} \right\} \ge \frac{{S\left( 0 \right)s^{\alpha - 1} }}{{s^{\alpha } + M_{1} }}. \\ \end{aligned}$$

By taking inverse Laplace transformation on both sides and by using the following result ${L}^{-1}\left\{\frac{{s}^{\alpha -\beta }}{{s}^{\alpha }-M}\right\}={t}^{\beta -1}{E}_{\alpha ,\beta }\left(M{t}^{\alpha }\right)$, we reach at the expression given below

$$S\left( t \right) \ge S\left( 0 \right)L^{ - 1} \left\{ {\frac{{s^{\alpha - 1} }}{{s^{\alpha } + M_{1} }}} \right\},$$

$$S\left( t \right) \ge S\left( 0 \right)E_{\alpha ,1} \left( { - M_{1} t^{\alpha } } \right),$$

$$S\left( t \right) \ge S\left( 0 \right)E_{\alpha } \left( { - M_{1} t^{\alpha } } \right),$$

$$S\left( t \right) \ge 0,{ }\forall t \ge 0.$$

Next, for the function I(t), we proceed as explained below

$${}_{0}^{c} D_{t}^{\alpha } I\left( t \right) = \frac{{\beta^{\alpha } SI}}{{1 + \alpha_{1}^{\alpha } I }} - \left( {\gamma^{\alpha } + \delta_{1}^{\alpha } + \mu^{\alpha } } \right)I,$$

$${}_{0}^{c} D_{t}^{\alpha } I\left( t \right) \ge - \left( {\gamma^{\alpha } + \delta_{1}^{\alpha } + \mu^{\alpha } } \right)I.$$

Let $$M_{2} = \gamma^{\alpha } + \delta_{1}^{\alpha } + \mu^{\alpha }$$, then

$${}_{0}^{c} D_{t}^{\alpha } I\left( t \right) \ge - M_{2} I,$$

$${}_{0}^{c} D_{t}^{\alpha } I\left( t \right) + M_{2} {\text{I}}\left( {\text{t}} \right) \ge 0.$$

By taking Laplace transformation on both sides, we derive the following result

$$L\{{}_{0}{}^{c}{D}_{t}^{\alpha }I\left(t\right)\}+{M}_{2}{\text{L}}\{{\text{I}}({\text{t}})\}\ge 0,$$

$${s}^{\alpha }L\left\{I\left(t\right)\right\}-{s}^{\alpha -1}I\left(0\right)+{M}_{2}L\left\{I\left(t\right)\right\}\ge 0,$$

$$({s}^{\alpha }+{M}_{2})L\left\{I\left(t\right)\right\}\ge {s}^{\alpha -1}I\left(0\right),$$

$$L\left\{I\left(t\right)\right\}\ge \frac{I(0){s}^{\alpha -1}}{{s}^{\alpha }+{M}_{2}}.$$

By taking inverse Laplace transformation on both sides and by using the following result

$${L}^{-1}\left\{\frac{{s}^{\alpha -\beta }}{{s}^{\alpha }-M}\right\}={t}^{\beta -1}{E}_{\alpha ,\beta }\left(M{t}^{\alpha }\right),$$

$$I(t)\ge {I(0)L}^{-1}\left\{\frac{{s}^{\alpha -1}}{{s}^{\alpha }+{M}_{2}}\right\},$$

$$I\left(t\right)\ge I\left(0\right){E}_{\alpha ,1}\left(-{M}_{2}{t}^{\alpha }\right),$$

$$I\left(t\right)\ge I\left(0\right){E}_{\alpha }\left(-{M}_{2}{t}^{\alpha }\right),$$

$$I\left(t\right)\ge 0, \forall t\ge 0.$$

Similarly, for the class V(t) we have

$${}_{0}{}^{c}{D}_{t}^{\alpha }V\left(t\right)={\delta }_{2}^{\alpha }S+{\delta }_{1}^{\alpha }I-({\sigma }^{\alpha }+{\mu }^{\alpha })V,$$

$${}_{0}{}^{c}{D}_{t}^{\alpha }V\left(t\right)\ge -({\sigma }^{\alpha }+{\mu }^{\alpha })V,$$

Let ${M}_{3}={\sigma }^{\alpha }+{\mu }^{\alpha }.$Then, we conclude that

$${}_{0}{}^{c}{D}_{t}^{\alpha }V\left(t\right)\ge -{M}_{3}V(t).$$

By solving above inequality, we get

$$V\left(t\right)\ge V\left(0\right){E}_{\alpha ,1}\left(-{M}_{3}{t}^{\alpha }\right),$$

$$V\left(t\right)\ge V\left(0\right){E}_{\alpha }\left(-{M}_{3}{t}^{\alpha }\right),$$

$$V\left(t\right)\ge 0, \forall t\ge 0.$$

Finally, for the function $R\left(t\right)$

$${}_{0}{}^{c}{D}_{t}^{\alpha }R\left(t\right)={\gamma }^{\alpha }I+{\sigma }^{\alpha }V-{\mu }^{\alpha }R,$$

$${}_{0}{}^{c}{D}_{t}^{\alpha }R\left(t\right)\ge -{\mu }^{\alpha }R,$$

By solving above inequality, we get

$$R\left(t\right)\ge R\left(0\right){E}_{\alpha ,1}\left(-{\mu }^{\alpha }{t}^{\alpha }\right),$$

$$R\left(t\right)\ge R\left(0\right){E}_{\alpha }\left(-{\mu }^{\alpha }{t}^{\alpha }\right),$$

$$R\left(t\right)\ge 0, \forall t\ge 0.$$

As desired.

Now, we present the second property of model (1) i.e. boundedness of the solution.

#### Theorem 2

(Boundedness) For any time t, the system (1) is bounded and lies in the feasible region $\Omega$.

#### Proof

By letting the population function $N(t)=S(t)+I(t)+V(t)+R(t)$, we advance as.

$${}_{0}{}^{c}{D}_{t}^{\alpha }N\left(t\right)={}_{0}{}^{c}{D}_{t}^{\alpha }S\left(t\right)+ {}_{0}{}^{c}{D}_{t}^{\alpha }I\left(t\right)+ {}_{0}{}^{c}{D}_{t}^{\alpha }V\left(t\right)+ {}_{0}{}^{c}{D}_{t}^{\alpha }R\left(t\right),$$

$${}_{0}{}^{c}{D}_{t}^{\alpha }N\left(t\right)=\Lambda -{\mu }^{\alpha }{\text{N}},$$

$${}_{0}{}^{c}{D}_{t}^{\alpha }N\left(t\right)+{\mu }^{\alpha }{\text{N}}({\text{t}}) =\Lambda .$$

By taking Laplace transform on both sides of the above expression

$$L\left\{{}_{0}{}^{c}{D}_{t}^{\alpha }N\left(t\right)\right\}+{\mu }^{\alpha }L\left\{{\text{N}}({\text{t}}) \right\}=\mathrm{\Lambda L}\left\{1\right\},$$

$$s^{\alpha } L\left\{ {{\text{N}}\left( {\text{t}} \right) } \right\} - s^{\alpha - 1} N\left( 0 \right) + { }\mu^{\alpha } L\left\{ {{\text{N}}\left( {\text{t}} \right) } \right\} = \frac{{\Lambda }}{s},$$

$$\left({s}^{\alpha }+{\mu }^{\alpha }\right)L\left\{{\text{N}}({\text{t}}) \right\}={s}^{\alpha -1}N\left(0\right)+\frac{\Lambda }{s},$$

$$L\left\{{\text{N}}({\text{t}}) \right\}=\frac{N(0){s}^{\alpha -1}}{{s}^{\alpha }+{\mu }^{\alpha }}+\frac{\Lambda }{s\left({s}^{\alpha }+{\mu }^{\alpha }\right)},$$

$$L\left\{{\text{N}}({\text{t}}) \right\}=N\left(0\right)\frac{{s}^{\alpha -1}}{{s}^{\alpha }+{\mu }^{\alpha }}+\Lambda \frac{{{\text{s}}}^{-1}}{{s}^{\alpha }+{\mu }^{\alpha }},$$

$$L\left\{{\text{N}}({\text{t}}) \right\}=N\left(0\right)\frac{{s}^{\alpha -1}}{{s}^{\alpha }+{\mu }^{\alpha }}+\Lambda \frac{{{\text{s}}}^{\mathrm{\alpha }-(1+\mathrm{\alpha })}}{{s}^{\alpha }+{\mu }^{\alpha }}.$$

By taking inverse Laplace transformation on both sides and by using the following formula ${L}^{-1}\left\{\frac{{s}^{\alpha -\beta }}{{s}^{\alpha }-M}\right\}={t}^{\beta -1}{E}_{\alpha ,\beta }\left(M{t}^{\alpha }\right)$, we get

$${\text{N}}\left({\text{t}}\right)=N\left(0\right){L}^{-1}\left\{\frac{{s}^{\alpha -1}}{{s}^{\alpha }+{\mu }^{\alpha }}\right\}+\Lambda {L}^{-1}\left\{\frac{{{\text{s}}}^{\mathrm{\alpha }-(1+\mathrm{\alpha })}}{{s}^{\alpha }+{\mu }^{\alpha }}\right\},$$

$$N\left(t\right)=N\left(0\right){E}_{\alpha ,1}\left(-{\mu }^{\alpha }{t}^{\alpha }\right)+\Lambda {t}^{\alpha } {E}_{\alpha ,\alpha +1}\left(-{\mu }^{\alpha }{t}^{\alpha }\right),$$

$$N\left(t\right)=N\left(0\right){E}_{\alpha ,1}\left(-{\mu }^{\alpha }{t}^{\alpha }\right)+{\mu }^{\alpha }.\frac{\Lambda }{{\mu }^{\alpha }}{t}^{\alpha } {E}_{\alpha ,\alpha +1}\left(-{\mu }^{\alpha }{t}^{\alpha }\right),$$

Let $$M=max\left\{N\left(0\right),\frac{\Lambda }{{\mu }^{\alpha }}\right\},$$

$$N\left(t\right)\le M\left\{{E}_{\alpha ,1}\left(-{\mu }^{\alpha }{t}^{\alpha }\right)+{\mu }^{\alpha }{t}^{\alpha } {E}_{\alpha ,\alpha +1}\left(-{\mu }^{\alpha }{t}^{\alpha }\right)\right\}.$$

By using, ${E}_{\alpha ,\beta }\left(z\right)=z{E}_{\alpha ,\alpha +\beta }\left(z\right)+\frac{1}{\Gamma (\upbeta )}$, we can write above inequality in the form

$$N\left(t\right)\le M\left\{-{\mu }^{\alpha }{t}^{\alpha } {E}_{\alpha ,\alpha +1}\left(-{\mu }^{\alpha }{t}^{\alpha }\right)+\frac{1}{\Gamma (1)}+{\mu }^{\alpha }{t}^{\alpha } {E}_{\alpha ,\alpha +1}\left(-{\mu }^{\alpha }{t}^{\alpha }\right)\right\},$$

$$N(t)\le M.$$

Thus $N(t)$ is bounded uniformly and hence the solution $X\left(t\right)=\left\{\left(S,I,V,R\right)\right\}$ of the system (1) is uniformly bounded in $\Omega =\left\{\left(S,I,V,R\right):S+I+V+R=N\le M\right\}$ for $t\in [0,\infty )$.

Therefore, the model (1) is well-posed, biologically and mathematically in the invariant set $\Omega$, as desired.

### Existence and uniqueness

This subsection presents the existence and uniqueness of solution of the proposed model using the technique of fixed-point theory, for this we will apply the following Lemma.

#### Lemma 1

(^39,46^).Consider the system ${}_{{t}_{0}}{}^{C}{D}_{t}^{a}\mathrm{ x}\left({\text{t}}\right)=\mathrm{ g}\left({\text{t}},\mathrm{ x}\right), {{\text{t}}}_{0} > 0,$ with initial condition ${\text{x}}\left({{\text{t}}}_{0}\right) = {{\text{x}}}_{{{\text{t}}}_{0}}$, where, $\mathrm{\alpha }\in \left(0, 1\right],\mathrm{ g}:\left[{{\text{t}}}_{0},\mathrm{ \infty }\right)\times\Omega \to {\mathbb{R}},\Omega \subseteq {{\text{C}}}^{1}[{t}_{0},\infty )$, if local Lipschitz condition is satisfied by *g*(*t*, *x*) with respect to *x*, then there exists a unique solution on $[{{\text{t}}}_{0},\mathrm{ \infty }) \times\Omega$*.*

#### Theorem 3

(Existence and Uniqueness) For any time t, the solution of system (1) will exist and the solution will be unique^47^.

#### Proof

To study the existence and uniqueness of system (1), let us consider the region $\Sigma \times [{{\text{t}}}_{0},\upgamma ]$, where.

$${\Sigma } = \left\{ {\left( {S,I,V,R} \right) \in {\mathbb{R}}^{4} , S,I,V,R \in C^{1} \left[ {t_{0} ,\infty } \right) \wedge \left\| S \right\|,\left\| I \right\|,\left\| V \right\|,\left\| R \right\| \le M} \right\}\quad {\text{and}}\quad {\gamma } < { } + \infty .$$

Let $$K\left(S\right)=\Lambda -\frac{{\beta }^{\alpha }SI}{1+{\alpha }_{1}^{\alpha }I }-\left({\delta }_{2}^{\alpha }+{\mu }^{\alpha }\right)S,$$

$$\begin{aligned} & \left\| {K\left( {S_{1} } \right) - K\left( {S_{2} } \right)} \right\| = \left\| {{\Lambda } - \frac{{\beta^{\alpha } S_{1} I}}{{1 + \alpha_{1}^{\alpha } I{ }}} - \left( {\delta_{2}^{\alpha } + \mu^{\alpha } } \right)S_{1} - {\Lambda } + \frac{{\beta^{\alpha } S_{2} I}}{{1 + \alpha_{1}^{\alpha } I{ }}} + \left( {\delta_{2}^{\alpha } + \mu^{\alpha } } \right)S_{2} } \right\|, \\ & = \left\| {\frac{{\beta^{\alpha } I}}{{1 + \alpha_{1}^{\alpha } I{ }}}\left( {S_{2} - S_{1} } \right) + \left( {\delta_{2}^{\alpha } + \mu^{\alpha } } \right)\left( {S_{2} - S_{1} } \right)} \right\|, \\ & \le \beta^{\alpha } \frac{1}{{1 + \alpha_{1}^{\alpha } I}}\left\| I \right\|\left\| { S_{2} - S_{1} } \right\| + \left( {\delta_{2}^{\alpha } + \mu^{\alpha } } \right)\left\| {S_{2} - S_{1} } \right\|, \\ & \le \beta^{\alpha } M \left\| {S_{2} - S_{1} } \right\| + \left( {\delta_{2}^{\alpha } + \mu^{\alpha } } \right)\left\| {S_{2} - S_{1} } \right\|,\quad {\text{where}}\quad \frac{I}{{1 + \alpha_{1}^{\alpha } I{ }}} \le 1, \\ & \le \left( {\beta^{\alpha } M + \delta_{2}^{\alpha } + \mu^{\alpha } } \right) \left\| {S_{2} - S_{1} } \right\|. \\ \end{aligned}$$

Therefore, $$\Vert K\left({S}_{1}\right)-K\left({S}_{2}\right)\Vert \le \left({\beta }^{\alpha }M+{\delta }_{2}^{\alpha }+{\mu }^{\alpha }\right)\Vert {S}_{1}{-S}_{2}\Vert .$$

Therefore, $K(S)$ satisfies Lipchitz condition.

For contraction mapping. ${\beta }^{\alpha }M+{\delta }_{2}^{\alpha }+{\mu }^{\alpha }<1,$

Let $$L\left(I\right)=\frac{{\beta }^{\alpha }SI}{1+{\alpha }_{1}^{\alpha }I }-\left({{\upgamma }^{\mathrm{\alpha }}+\delta }_{1}^{\alpha }+{\mu }^{\alpha }\right)I,$$

$$\begin{aligned} \left\| {L\left( {I_{1} } \right) - L\left( {I_{2} } \right)} \right\| & = \left\| {\frac{{\beta^{\alpha } SI_{1} }}{{1 + \alpha_{1}^{\alpha } I_{1} { }}} - \left( {{\upgamma }^{{\upalpha }} + \delta_{1}^{\alpha } + \mu^{\alpha } } \right)I_{1} - \frac{{\beta^{\alpha } SI_{2} }}{{1 + \alpha_{1}^{\alpha } I_{2} { }}} + \left( {{\upgamma }^{{\upalpha }} + \delta_{1}^{\alpha } + \mu^{\alpha } } \right)I_{2} } \right\|, \\ & = \left\| {\beta^{\alpha } S\left( {\frac{{I_{1} }}{{1 + \alpha_{1}^{\alpha } I_{1} { }}} - \frac{{I_{2} }}{{1 + \alpha_{1}^{\alpha } I_{2} { }}}} \right) + \left( {{\upgamma }^{{\upalpha }} + \delta_{1}^{\alpha } + \mu^{\alpha } } \right)\left( {I_{2} - I_{1} } \right)} \right\|, \\ & \le \beta^{\alpha } \left\| S \right\|\left\| {\frac{{I_{1} + \alpha_{1}^{\alpha } I_{1} I_{2} - I_{2} - \alpha_{1}^{\alpha } I_{1} I_{2} }}{{\left( {1 + \alpha_{1}^{\alpha } I_{1} } \right)\left( {1 + \alpha_{1}^{\alpha } I_{2} } \right)}}} \right\| + \left( {{\upgamma }^{{\upalpha }} + \delta_{1}^{\alpha } + \mu^{\alpha } } \right)\left\| {I_{2} - I_{1} } \right\|, \\ & \le \beta^{\alpha } M \cdot \frac{1}{{1 + \alpha_{1}^{\alpha } I_{1} { }}} \cdot \frac{1}{{1 + \alpha_{1}^{\alpha } I_{2} { }}} \cdot \left\| {I_{1} - I_{2} } \right\| + \left( {{\upgamma }^{{\upalpha }} + \delta_{1}^{\alpha } + \mu^{\alpha } } \right)\left\| {I_{2} - I_{1} } \right\|, \\ & \le \beta^{\alpha } MI_{1} - I_{2} + \left( {{\upgamma }^{{\upalpha }} + \delta_{1}^{\alpha } + \mu^{\alpha } } \right)I_{2} - I_{1} ,\quad {\text{where}}\quad \frac{I}{{1 + \alpha_{1}^{\alpha } I{ }}} \le 1,\frac{{\overline{I}}}{{1 + \alpha_{1}^{\alpha } \overline{I}{ }}} \le 1, \\ & \le \left( {\beta^{\alpha } M + {\upgamma }^{{\upalpha }} + \delta_{1}^{\alpha } + \mu^{\alpha } } \right)\left\| {I_{1} - I_{2} } \right\|. \\ \end{aligned}$$

Therefore, $$\Vert L\left({I}_{1}\right)-L\left({I}_{2}\right)\Vert \le \left({\beta }^{\alpha }M+{{\upgamma }^{\mathrm{\alpha }}+\delta }_{1}^{\alpha }+{\mu }^{\alpha }\right)\Vert {I}_{1}-{I}_{2}\Vert$$.

Consequently, $L(I)$ satisfies Lipchitz condition.

For contraction mapping, ${\beta }^{\alpha }M+{{\upgamma }^{\mathrm{\alpha }}+\delta }_{1}^{\alpha }+{\mu }^{\alpha }<1.$

Let $$N\left(V\right)={\delta }_{2}^{\alpha }S+{\delta }_{1}^{\alpha }I-\left({\sigma }^{\alpha }+{\mu }^{\alpha }\right)V,$$

$$\begin{aligned} \left\| {N\left( {V_{1} } \right) - N\left( {V_{2} } \right)} \right\| & = \left\| {\delta_{2}^{\alpha } S + \delta_{1}^{\alpha } I - \left( {\sigma^{\alpha } + \mu^{\alpha } } \right)V_{1} - \delta_{2}^{\alpha } S - \delta_{1}^{\alpha } I + \left( {\sigma^{\alpha } + \mu^{\alpha } } \right)V_{2} } \right\|, \\ & = \left\| {\left( {\sigma^{\alpha } + \mu^{\alpha } } \right)\left( {V_{2} - V_{1} } \right)} \right\|, \\ & = \left| {\sigma^{\alpha } + \mu^{\alpha } } \right|\left\| {V_{2} - V_{1} } \right\|, \\ & \le \left( {\left| {\sigma^{\alpha } } \right| + \left| {\mu^{\alpha } } \right|} \right)\left\| {V_{1} - V_{2} } \right\|, \\ & \le \left( {\sigma^{\alpha } + \mu^{\alpha } } \right)\left\| {V_{1} - V_{2} } \right\|. \\ \end{aligned}$$

Therefore, $$\Vert N\left({V}_{1}\right)-N\left({V}_{2}\right)\Vert <\left({\sigma }^{\alpha }+{\mu }^{\alpha }\right)\Vert {V}_{1}-{V}_{2}\Vert$$.

So, $N(V)$ satisfies Lipchitz condition.

For contraction mapping, ${\sigma }^{\alpha }+{\mu }^{\alpha }<1$.

Let $$P\left(R\right)={\gamma }^{\alpha }I+{\sigma }^{\alpha }V-{\mu }^{\alpha }R.$$

$$\begin{aligned} \left\| {P\left( {R_{1} } \right) - P\left( {R_{2} } \right)} \right\| & = \left\| {\gamma^{\alpha } I + \sigma^{\alpha } V - \mu^{\alpha } R_{1} - \gamma^{\alpha } I - \sigma^{\alpha } V + \mu^{\alpha } R_{2} } \right\|, \\ & = \mu^{\alpha } \left\| {R_{2} - R_{1} } \right\|, \\ & = \mu^{\alpha } \left\| {R_{1} - R_{2} } \right\|. \\ \end{aligned}$$

$$\Vert P\left({R}_{1}\right)-P\left({R}_{2}\right)\Vert <\Vert {R}_{1}-{R}_{2}\Vert .$$ Where $${\mu }^{\alpha }<1$$.

Therefore, $P(R)$ satisfies Lipchitz condition.

Let, $${F}_{1}={\beta }^{\alpha }M+{\delta }_{2}^{\alpha }+{\mu }^{\alpha }$$, $${F}_{2}={\beta }^{\alpha }M+{{\upgamma }^{\mathrm{\alpha }}+\delta }_{1}^{\alpha }+{\mu }^{\alpha }$$, $${F}_{3}={\sigma }^{\alpha }+{\mu }^{\alpha }$$ , $${F}_{4}={\mu }^{\alpha }$$.

Also let $$F=max\left\{{F}_{1},{F}_{2},{F}_{3},{F}_{4}\right\}$$.

Therefore,

$$\Vert K\left({S}_{1}\right)-K\left({S}_{2}\right)\Vert \le F\Vert {S}_{1}{-S}_{2}\Vert ,$$

$$\Vert L\left({I}_{1}\right)-L\left({I}_{2}\right)\Vert \le F\Vert {I}_{1}-{I}_{2}\Vert ,$$

$$\Vert N\left({V}_{1}\right)-N\left({V}_{2}\right)\Vert \le {\text{F}}\Vert {V}_{1}-{V}_{2}\Vert ,$$

$$\Vert P\left({R}_{1}\right)-P\left({R}_{2}\right)\Vert \le F\Vert {R}_{1}-{R}_{2}\Vert .$$

For $F<1,$ $K(S), L(I), N(V), P(R)$ are contraction mappings.

Therefore, $K(S), L(I), N(V)$ and $P(R)$ satisfies Lipshitz conditions and are contraction mappings. Therefore, according to Banach fixed point theorem, solution of the proposed model (1) exists and is unique, as desired.

## Qualitative analysis of the proposed model

This section presents the equilibrium points, basic reproductive number and stability analysis, analytically. The order of the results for the stability is described as:

- Asymptotically local stability at corona virus-free equilibrium is established by Theorem 4.
- Asymptotically local stability at corona existing equilibrium is ensured by Theorem 5.
- Asymptotically global stability at corona virus-free equilibrium is guaranteed by Theorem 6.
- Asymptotically global stability at corona existing equilibrium is confirmed by Theorem 7.

### Equilibria

We determine the equilibria of the system (1) by assuming the state variables are constant and by putting the right side equal to zero. Equation (1) admits two types of equilibria as follows:

(i) corona virus-free equilibrium = ${C}_{0}=\left({S}_{0},{I}_{0},{V}_{0},{R}_{0}\right)=\left(\frac{\Lambda }{{\delta }_{2}^{\alpha }+{\mu }^{\alpha }},\mathrm{0,0},0\right)$,
(ii) corona existing equilibrium = ${C}_{1}=\left({S}^{*},{I}^{*},{V}^{*},{R}^{*}\right)$,
  $$\begin{aligned} {\text{where}}\quad S^{*} & = \frac{{\left( {\gamma^{\alpha } + \delta_{1}^{\alpha } + \mu^{\alpha } } \right)\left( {1 + \alpha^{\alpha } I^{*} } \right)}}{{\beta^{\alpha } }}, \\ I^{*} & = \frac{{{\upbeta }^{\alpha } {\Lambda } - \left( {\delta_{2}^{\alpha } + \mu^{\alpha } } \right)\left( {\delta_{1}^{\alpha } + \mu^{\alpha } } \right)}}{{\beta^{\alpha } \left( {\delta_{1}^{\alpha } + \mu^{\alpha } } \right) + \alpha^{\alpha } \left( {\delta_{2}^{\alpha } + \mu^{\alpha } } \right)\left( {\delta_{1}^{\alpha } + \mu^{\alpha } } \right)}}, \\ V^{*} & = \frac{{\delta_{2}^{\alpha } S^{*} + \delta_{1}^{\alpha } I^{*} }}{{\sigma^{\alpha } + \mu^{\alpha } }},\quad R^{*} = \frac{{\gamma^{\alpha } I^{*} + \sigma^{\alpha } V^{*} }}{{\mu^{\alpha } }}. \\ \end{aligned}$$

### Reproduction number

We determine the reproduction number of the system (1) by using the well-known results like the next-generation matrix method after substituting the value of coronavirus free equilibrium. We get:

$$\left[\begin{array}{c}I{\prime}\\ V{\prime}\\ R{\prime}\end{array}\right]=\left[\begin{array}{ccc}\frac{{\upbeta }^{\mathrm{\alpha }}\Lambda }{{\delta }_{2}^{\mathrm{\alpha }}+{\mu }^{\mathrm{\alpha }}}& 0& 0\\ 0& 0& 0\\ 0& 0& 0\end{array}\right]\left[\begin{array}{c}I\\ V\\ R\end{array}\right]-\left[\begin{array}{ccc}{{\gamma }^{\mathrm{\alpha }}+\delta }_{1}^{\mathrm{\alpha }}+{\mu }^{\mathrm{\alpha }}& 0& 0\\ -{\delta }_{1}^{\mathrm{\alpha }}({\sigma }^{\mathrm{\alpha }}+{\mu }^{\mathrm{\alpha }})& 0& 0\\ -{\gamma }^{\mathrm{\alpha }}& -{\sigma }^{\mathrm{\alpha }}& {\mu }^{\mathrm{\alpha }}\end{array}\right]\left[\begin{array}{c}I\\ V\\ R\end{array}\right]$$

where $$A=\left[\begin{array}{ccc}\frac{{\upbeta }^{\mathrm{\alpha }}\Lambda }{{\delta }_{2}^{\mathrm{\alpha }}+{\mu }^{\mathrm{\alpha }}}& 0& 0\\ 0& 0& 0\\ 0& 0& 0\end{array}\right] , B=\left[\begin{array}{ccc}{{\gamma }^{\mathrm{\alpha }}+\delta }_{1}^{\mathrm{\alpha }}+{\mu }^{\mathrm{\alpha }}& 0& 0\\ -{\delta }_{1}^{\mathrm{\alpha }}({\sigma }^{\mathrm{\alpha }}+{\mu }^{\mathrm{\alpha }})& 0& 0\\ -{\gamma }^{\mathrm{\alpha }}& -{\sigma }^{\mathrm{\alpha }}& {\mu }^{\mathrm{\alpha }}\end{array}\right],$$

$$A{B}^{-1}=\left[\begin{array}{ccc}\frac{{\upbeta }^{\mathrm{\alpha }}\Lambda }{\left({\delta }_{2}^{\mathrm{\alpha }}+{\mu }^{\mathrm{\alpha }}\right)\left({{\gamma }^{\mathrm{\alpha }}+\delta }_{1}^{\mathrm{\alpha }}+{\mu }^{\mathrm{\alpha }}\right)}& 0& 0\\ 0& 0& 0\\ 0& 0& 0\end{array}\right],$$

${R}_{0}$ is the spectral radius of $A{B}^{-1}$.

Mathematically ${R}_{0}$ is expressed by the following parametric relation

$${R}_{0}=\frac{{\upbeta }^{\mathrm{\alpha }}\Lambda }{\left({\delta }_{2}^{\mathrm{\alpha }}+{\mu }^{\mathrm{\alpha }}\right)\left({\gamma }^{\mathrm{\alpha }}+{\delta }_{1}^{\mathrm{\alpha }}+{\mu }^{\mathrm{\alpha }}\right)}.$$

### Stability analysis

In this section, we test the local and global stability of the system (1), considering the two defined equilibria.

#### Theorem 4

(Local stability at ${{\text{C}}}_{0})$ The system (1) at ${{\text{C}}}_{0}=\left({S}_{0},{I}_{0},{V}_{0},{R}_{0}\right)=\left(\frac{\Lambda }{{{\varvec{\delta}}}_{2}^{\mathrm{\alpha }}+{{\varvec{\mu}}}^{\mathrm{\alpha }}},\mathrm{0,0},0\right)$ is locally asymptotically stable if ${R}_{0}<1$. Otherwise, unstable when ${R}_{0}>1$.

#### Proof

The Jacobian matrix is obtained for the system (1) as follows.

$$J\left(S,I,V,R\right)=\left[\begin{array}{cccc}-\frac{{\beta }^{\alpha }I}{1+{\alpha }_{1}^{\alpha }I}-{{\varvec{\delta}}}_{2}^{\alpha }-{{\varvec{\mu}}}^{\alpha }& -\frac{{\beta }^{\alpha }S}{{\left(1+{\alpha }_{1}^{\alpha }I\right)}^{2}}& 0& 0\\ \frac{{\beta }^{\alpha }I}{1+{\alpha }_{1}^{\alpha }I}& \frac{{\beta }^{\alpha }S}{{\left(1+{\alpha }_{1}^{\alpha }I\right)}^{2}}-{\gamma }^{\alpha }-{\delta }_{1}^{\alpha }-{\mu }^{\alpha }& 0& 0\\ {\delta }_{2}^{\alpha }& {\delta }_{1}^{\alpha }& -{\sigma }^{\alpha }-{\mu }^{\alpha }& 0\\ 0& {\gamma }^{\alpha }& {\sigma }^{\alpha }& -{\mu }^{\alpha }\end{array}\right].$$

The Jacobian matrix at ${C}_{0}$ is computed as follows

$$J\left(\frac{\Lambda }{{\delta }_{2}^{\mathrm{\alpha }}+{\mu }^{\mathrm{\alpha }}},\mathrm{0,0},0\right)=\left[\begin{array}{cccc}-{\delta }_{2}^{\mathrm{\alpha }}-{\mu }^{\alpha }& -\frac{{\upbeta }^{\alpha }\Lambda }{{\delta }_{2}^{\mathrm{\alpha }}+{\mu }^{\alpha }}& 0& 0\\ \frac{{\beta }^{\alpha }I}{1+{\mathrm{\alpha }}_{1}^{\mathrm{\alpha }}{\text{I}}}& \frac{{\upbeta }^{\alpha }\Lambda }{{\delta }_{2}^{\alpha }+{\mu }^{\alpha }}-{\gamma }^{\mathrm{\alpha }}-{\delta }_{1}^{\mathrm{\alpha }}-{\mu }^{\alpha }& 0& 0\\ {\delta }_{2}^{\alpha }& {\delta }_{1}^{\alpha }& -{\sigma }^{\alpha }-{\mu }^{\alpha }& 0\\ 0& {\gamma }^{\alpha }& {\sigma }^{\alpha }& -{\mu }^{\alpha }\end{array}\right].$$

Consider $\left|J-\lambda I\right|=0$, and hence

$$\left|\begin{array}{cccc}-{\delta }_{2}^{\alpha }-{\mu }^{\alpha }-\lambda & -\frac{{\upbeta }^{\alpha }\Lambda }{{\delta }_{2}^{\alpha }+{\mu }^{\alpha }}& 0& 0\\ \frac{{\beta }^{\alpha }I}{1+{\mathrm{\alpha }}_{1}^{\mathrm{\alpha }}{\text{I}}I}& \frac{{\upbeta }^{\alpha }\Lambda }{{\delta }_{2}^{\alpha }+{\mu }^{\alpha }}-{\gamma }^{\mathrm{\alpha }}-{\delta }_{1}^{\mathrm{\alpha }}-{\mu }^{\alpha }-\lambda & 0& 0\\ {\delta }_{2}^{\alpha }& {\delta }_{1}^{\alpha }& -{\sigma }^{\alpha }-{\mu }^{\alpha }-\lambda & 0\\ 0& {\gamma }^{\alpha }& {\sigma }^{\alpha }& -{\mu }^{\alpha }-\lambda \end{array}\right|=0.$$

After solving above determinant, we get ${\lambda }_{1}=-{\mu }^{\alpha }<0 , {\lambda }_{2}=-\left({\sigma }^{\alpha }+{\mu }^{\alpha }\right)<0,$

$${\lambda }_{3}=-\left({\delta }_{2}^{\alpha }+{\mu }^{\alpha }\right)<0 {\lambda }_{4}=\frac{{\upbeta }^{\alpha }\Lambda }{{\delta }_{2}^{\alpha }+{\mu }^{\alpha }}-{\gamma }^{\mathrm{\alpha }}-{\delta }_{1}^{\mathrm{\alpha }}-{\mu }^{\alpha }<0.$$

Consequently, we draw the following result

$$\frac{{\upbeta }^{\mathrm{\alpha }}\Lambda }{\left({\delta }_{2}^{\mathrm{\alpha }}+{\mu }^{\mathrm{\alpha }}\right)\left({\gamma }^{\mathrm{\alpha }}+{\delta }_{1}^{\mathrm{\alpha }}+{\mu }^{\alpha }\right)}<1 , {R}_{0}<1.$$

It is clear that, system (1) is locally asymptotically stable at ${C}_{0}$ when ${R}_{0}<1,$ as desired.

#### Theorem 5

(Local stability at ${{\text{C}}}_{1})$ The system (1) at ${C}_{1}=\left({S}^{*},{I}^{*},{V}^{*},{R}^{*}\right)$ is locally asymptotically stable if ${R}_{0}>1$.

#### Proof

The Jacobian matrix at ${C}_{1}$ of the system (1) is as follows.

$$J\left({S}^{*},{I}^{*},{V}^{*},{R}^{*}\right)=\left[\begin{array}{cccc}-\frac{{\beta }^{\alpha }{I}^{*}}{1+{\mathrm{\alpha }}_{1}^{\mathrm{\alpha }}{I}^{*}}-{\delta }_{2}^{\mathrm{\alpha }}-{\mu }^{\alpha }& -\frac{{\beta }^{\alpha }{S}^{*}}{{\left(1+{\mathrm{\alpha }}_{1}^{\mathrm{\alpha }}{I}^{*}\right)}^{2}}& 0& 0\\ \frac{{\beta }^{\alpha }{I}^{*}}{1+{\mathrm{\alpha }}_{1}^{\mathrm{\alpha }}{I}^{*}}& \frac{{\upbeta }^{\alpha }{{\text{S}}}^{*}}{{\left(1+{\mathrm{\alpha }}_{1}^{\mathrm{\alpha }}{I}^{*}\right)}^{2}}-{\gamma }^{\mathrm{\alpha }}-{\delta }_{1}^{\mathrm{\alpha }}-{\mu }^{\alpha }& 0& 0\\ {\delta }_{2}^{\alpha }& {\delta }_{1}^{\alpha }& -{\sigma }^{\alpha }-{\mu }^{\alpha }& 0\\ 0& {\gamma }^{\alpha }& {\sigma }^{\alpha }& -{\mu }^{\alpha }\end{array}\right].$$

Consider $\left|J-\lambda I\right|=0$, and hence

$$\left|\begin{array}{cccc}-\frac{{\beta }^{\alpha }{I}^{*}}{1+{\mathrm{\alpha }}_{1}^{\mathrm{\alpha }}{I}^{*}}-{\delta }_{2}^{\alpha }-{\mu }^{\alpha }-\lambda & -\frac{{\beta }^{\alpha }{S}^{*}}{{\left(1+{\mathrm{\alpha }}_{1}^{\mathrm{\alpha }}{I}^{*}\right)}^{2}}& 0& 0\\ \frac{{\beta }^{\alpha }{I}^{*}}{1+{\mathrm{\alpha }}_{1}^{\mathrm{\alpha }}{I}^{*}}& \frac{{\upbeta }^{\alpha }{{\text{S}}}^{*}}{{\left(1+{\mathrm{\alpha }}_{1}^{\mathrm{\alpha }}{ I}^{*}\right)}^{2}}-{\gamma }^{\alpha }-{\delta }_{1}^{\mathrm{\alpha }}-{\mu }^{\alpha }-\lambda & 0& 0\\ {\delta }_{2}^{\alpha }& {\delta }_{1}^{\alpha }& -{\sigma }^{\alpha }-{\mu }^{\alpha }-\lambda & 0\\ 0& {\gamma }^{\alpha }& {\sigma }^{\alpha }& -{\mu }^{\alpha }-\lambda \end{array}\right|=0.$$

After solving above determinant, ${\lambda }_{1}=-{\mu }^{\alpha }<0 , {\lambda }_{2}=-\left({\sigma }^{\alpha }+{\mu }^{\alpha }\right)<0,$

$$\begin{aligned} & \lambda^{2} + \left( {\frac{{\beta^{\alpha } I^{*} }}{{1 + {\upalpha }_{1}^{{\upalpha }} I^{*} }} - \frac{{{\upbeta }^{\alpha } {\text{S}}^{*} }}{{\left( {1 + {\upalpha }_{1}^{{\upalpha }} I^{*} } \right)^{2} }} + \gamma^{\alpha } + \delta_{1}^{\alpha } + \delta_{2}^{\alpha } + 2\mu^{\alpha } } \right) \\ & \lambda + \left( {\gamma^{\alpha } + \delta_{1}^{\alpha } + \mu^{\alpha } } \right)\left( {\frac{{\beta^{\alpha } I^{*} }}{{1 + {\upalpha }_{1}^{{\upalpha }} I^{*} }} + \delta_{2}^{\alpha } + \mu^{\alpha } } \right) - \frac{{\left( {\delta_{2}^{\alpha } + \mu^{\alpha } } \right){\upbeta }^{\alpha } {\text{S}}^{*} }}{{\left( {1 + {\upalpha }_{1}^{{\upalpha }} I^{*} } \right)^{2} }} = 0, \\ \end{aligned}$$

$${\lambda }^{2}+{A}_{1}\lambda +{A}_{0}=0.$$

where $${A}_{1}=\frac{{\beta }^{\alpha }{I}^{*}}{1+{\mathrm{\alpha }}_{1}^{\mathrm{\alpha }}{I}^{*}}-\frac{{\upbeta }^{\alpha }{{\text{S}}}^{*}}{{\left(1+{\mathrm{\alpha }}_{1}^{\mathrm{\alpha }}{I}^{*}\right)}^{2}}+{\gamma }^{\alpha }+{\delta }_{1}^{\alpha }+ {\delta }_{2}^{\alpha }+2{\mu }^{\alpha },$$

$${A}_{0}=\left({{\gamma }^{\alpha }+\delta }_{1}^{\alpha }+{\mu }^{\alpha }\right)\left(\frac{{\beta }^{\alpha }{I}^{*}}{1+{\mathrm{\alpha }}_{1}^{\mathrm{\alpha }}{I}^{*}}+{\delta }_{2}^{\alpha }+{\mu }^{\alpha }\right)-\frac{\left({\delta }_{2}^{\alpha }+{\mu }^{\alpha }\right){\upbeta }^{\alpha }{{\text{S}}}^{*}}{{\left(1+{\mathrm{\alpha }}_{1}^{\mathrm{\alpha }}{I}^{*}\right)}^{2}}.$$

Since, ${A}_{1}, {A}_{0}$ both are positive when ${R}_{0}>1$, by the Routh-Hurwitz criterion for the second order polynomial, the system (1) is locally asymptotically stable at ${C}_{1}$ when ${R}_{0}>1,$ as desired.

The following lemma is provided to improve the global stability analysis of the system.

#### Lemma 2

(^25^) Let $x:[0,\infty )\to {\mathbb{R}}^{+}$ be a continuous function and let ${t}_{0}\ge 0$. Then, for any time $t\ge {t}_{0} , \alpha \in \left(\mathrm{0,1}\right) and {x}^{*}\in {\mathbb{R}}^{+} ,$ the following inequality holds.

$${}_{0}{}^{c}{D}_{t}^{\alpha }\left[x\left(t\right)- {x}^{*}- {x}^{*}ln \frac{x(t)}{ {x}^{*}}\right]\le \left(1-\frac{{x}^{*}(t)}{x}\right) {}_{0}{}^{c}{D}_{t}^{\alpha }x(t).$$

We tackle now the global asymptotic stability of the system at the equilibrium points^46^.

#### Theorem 6

(Global stability at ${{\text{C}}}_{0})$ The system (1) at ${{\text{C}}}_{0}=\left({S}_{0},{I}_{0},{V}_{0},{R}_{0}\right)=\left(\frac{\Lambda }{{\delta }_{2}^{\mathrm{\alpha }}+{\mu }^{\mathrm{\alpha }}},\mathrm{0,0},0\right)$ is globally asymptotically stable if ${R}_{0}<1$.

#### Proof

Firstly, we define the Lyapunov function as.

$$L=\left(S+(I+V+R)\right)-{S}_{0}-{S}_{0}log \frac{S}{{S}_{0}},$$

$$L=\left(S-{S}_{0}-{S}_{0}log \frac{S}{{S}_{0}}\right)+(I+V+R).$$

Apply Caputo fractional derivative on both the sides,

$${}_{0}{}^{c}{D}_{t}^{\alpha }L={}_{0}{}^{c}{D}_{t}^{\alpha }\left(S-{S}_{0}-{S}_{0}log \frac{S}{{S}_{0}}\right)+\left({}_{0}{}^{c}{D}_{t}^{\alpha }I+{}_{0}{}^{c}{D}_{t}^{\alpha }V+{}_{0}{}^{c}{D}_{t}^{\alpha }R\right).$$

By using Lemma 1, following result is obtained

$${}_{0}{}^{c}{D}_{t}^{\alpha }L\le \left(1-\frac{{S}_{0}}{S}\right){}_{0}{}^{c}{D}_{t}^{\alpha }S+{}_{0}{}^{c}{D}_{t}^{\alpha }I+{}_{0}{}^{c}{D}_{t}^{\alpha }V+{}_{0}{}^{c}{D}_{t}^{\alpha }R,$$

$${}_{0}{}^{c}{D}_{t}^{\alpha }L\le \left(\frac{{S-S}_{0}}{S}\right)\left[\Lambda -\left(\frac{{\beta }^{\alpha }I}{1+{\alpha }_{1}^{\alpha }I }+{\delta }_{2}^{\alpha }+{\mu }^{\alpha }\right)\mathrm{S }\right]+\frac{{\beta }^{\alpha }SI}{1+{\alpha }_{1}^{\alpha }I }-\left({\gamma }^{\alpha }+{\delta }_{1}^{\alpha }+{\mu }^{\alpha }\right)I+{\delta }_{2}^{\alpha }S+{\delta }_{1}^{\alpha }I-\left({\sigma }^{\alpha }+{\mu }^{\alpha }\right)V+{\gamma }^{\alpha }I+{\sigma }^{\alpha }V-{\mu }^{\alpha }R,$$

$${}_{0}{}^{c}{D}_{t}^{\alpha }L\le \left(\frac{{S-S}_{0}}{S}\right)\left[\Lambda -\left(\frac{{\beta }^{\alpha }I}{1+{\alpha }_{1}^{\alpha }I }+{\delta }_{2}^{\alpha }+{\mu }^{\alpha }\right)\mathrm{S }\right]+\left({\gamma }^{\alpha }+{\delta }_{1}^{\alpha }+{\mu }^{\alpha }\right)\left(\frac{{\beta }^{\alpha }S}{\left(1+{\alpha }_{1}^{\alpha }I\right)\left({\gamma }^{\alpha }+{\delta }_{1}^{\alpha }+{\mu }^{\alpha }\right)}-1\right)I$$

$$-\left({\sigma }^{\alpha }+{\mu }^{\alpha }\right)\left(V-\frac{{\delta }_{2}^{\alpha }S+{\delta }_{1}^{\alpha }I}{{\sigma }^{\alpha }+{\mu }^{\alpha }}\right)-{\mu }^{\alpha }\left(R-\frac{{\gamma }^{\alpha }I+{\sigma }^{\alpha }V}{{\mu }^{\alpha }}\right),$$

$${}_{0}{}^{c}{D}_{t}^{\alpha }L\le \left(\frac{{S-S}_{0}}{S}\right)\left[\Lambda -\frac{\mathrm{\Lambda S}}{{S}_{0}}\right]+\left({\gamma }^{\alpha }+{\delta }_{1}^{\alpha }+{\mu }^{\alpha }\right)\left(\frac{{\beta }^{\alpha }\Lambda }{\left({\alpha }_{2}^{\alpha }+{\mu }^{\alpha }\right)\left({\gamma }^{\alpha }+{\delta }_{1}^{\alpha }+{\mu }^{\alpha }\right)}-1\right)I$$

$$-\left({\sigma }^{\alpha }+{\mu }^{\alpha }\right)\left(V-\frac{{\delta }_{2}^{\alpha }S+{\delta }_{1}^{\alpha }I}{{\sigma }^{\alpha }+{\mu }^{\alpha }}\right)-{\mu }^{\alpha }\left(R-\frac{{\gamma }^{\alpha }I+{\sigma }^{\alpha }V}{{\mu }^{\alpha }}\right),$$

$${}_{0}{}^{c}{D}_{t}^{\alpha }L\le -\frac{\Lambda {\left({S-S}_{0}\right)}^{2}}{S{S}_{0}}+\left({\gamma }^{\alpha }+{\delta }_{1}^{\alpha }+{\mu }^{\alpha }\right)\left({R}_{0}-1\right)I-\left({\sigma }^{\alpha }+{\mu }^{\alpha }\right)\left(V-\frac{{\delta }_{2}^{\alpha }S+{\delta }_{1}^{\alpha }I}{{\sigma }^{\alpha }+{\mu }^{\alpha }}\right)$$

$$-{\mu }^{\alpha }\left(R-\frac{{\gamma }^{\alpha }I+{\sigma }^{\alpha }V}{{\mu }^{\alpha }}\right).$$

Observe that ${}_{0}{}^{c}{D}_{t}^{\alpha }L<0$ when ${R}_{0}<1.$

Hence, the system is globally asymptotically stable at the disease-free equilibrium ${{\text{C}}}_{0}$.

#### Theorem 7

(Global stability at ${{\text{C}}}_{1})$ The system (1) at ${{\text{C}}}_{1}=\left({{\text{S}}}^{*},{{{\text{I}}}^{*},\mathrm{ V}}^{*},{{\text{R}}}^{*}\right)$ is globally asymptotically stable if ${R}_{0}>1$.

#### Proof

The proof of this theorem is similar to the previous theorem. To prove this theorem, we construct the Lyapunov functional at ${C}_{1}$ as:

$$L=\left(S-{S}^{*}-{S}^{*}log \frac{S}{{S}^{*}}\right)+\left(I-{I}^{*}-{I}^{*}log \frac{I}{{I}^{*}}\right)+\left(V-{V}^{*}-{V}^{*}log \frac{V}{{V}^{*}}\right)+\left(R-{R}^{*}-{R}^{*}log \frac{S}{{R}^{*}}\right),$$

$${}_{0}{}^{c}{D}_{t}^{\alpha }L={}_{0}{}^{c}{D}_{t}^{\alpha }\left(S-{S}^{*}-{S}^{*}log \frac{S}{{S}^{*}}\right)+{}_{0}{}^{c}{D}_{t}^{\alpha }\left(I-{I}^{*}-{I}^{*}log \frac{I}{{I}^{*}}\right)+{}_{0}{}^{c}{D}_{t}^{\alpha }\left(V-{V}^{*}-{V}^{*}log \frac{V}{{V}^{*}}\right)+{}_{0}{}^{c}{D}_{t}^{\alpha }\left(R-{R}^{*}-{R}^{*}log \frac{S}{{R}^{*}}\right).$$

By using Lemma 1:

$${}_{0}{}^{c}{D}_{t}^{\alpha }L\le \left(\frac{S-{S}^{*}}{S}\right){}_{0}{}^{c}{D}_{t}^{\alpha }S+\left(\frac{I-{I}^{*}}{I}\right){}_{0}{}^{c}{D}_{t}^{\alpha }I+\left(\frac{V-{V}^{*}}{V}\right){}_{0}{}^{c}{D}_{t}^{\alpha }V+\left(\frac{R-{R}^{*}}{R}\right){}_{0}{}^{c}{D}_{t}^{\alpha }R.$$

After some simplifications, we get:

$${}_{0}{}^{c}{D}_{t}^{\alpha }L\le -\frac{\Lambda {\left(S-{S}^{*}\right)}^{2}}{S{S}^{*}}-{\alpha }_{1}^{\alpha }{\beta }^{\alpha }S\frac{{\left(I-{I}^{*}\right)}^{2}}{\left(1+{\alpha }_{1}^{\alpha }I\right)(1+{\alpha }_{1}^{\alpha }{I}^{*})}-\left({\delta }_{2}^{\alpha }S+{\delta }_{1}^{\alpha }I\right)\frac{{\left(V-{V}^{*}\right)}^{2}}{V{V}^{*}}-\left({\gamma }^{\alpha }I+{\sigma }^{\alpha }V\right)\frac{{\left(R-{R}^{*}\right)}^{2}}{R{R}^{*}}.$$

Observe that ${}_{0}{}^{c}{D}_{t}^{\alpha }L\le 0$ when ${R}_{0}>1.$ Moreover, ${}_{0}{}^{c}{D}_{t}^{\alpha }L=0$ if $S={S}^{*}, I={I}^{*}, V={V}^{*}, R={R}^{*}.$

Therefore, the system is globally asymptotically stable at the endemic equilibrium ${{\text{C}}}_{1}$ , as desired.

## Numerical simulations

In this section, parametric values for the simulations are described.

**Figure 2:**
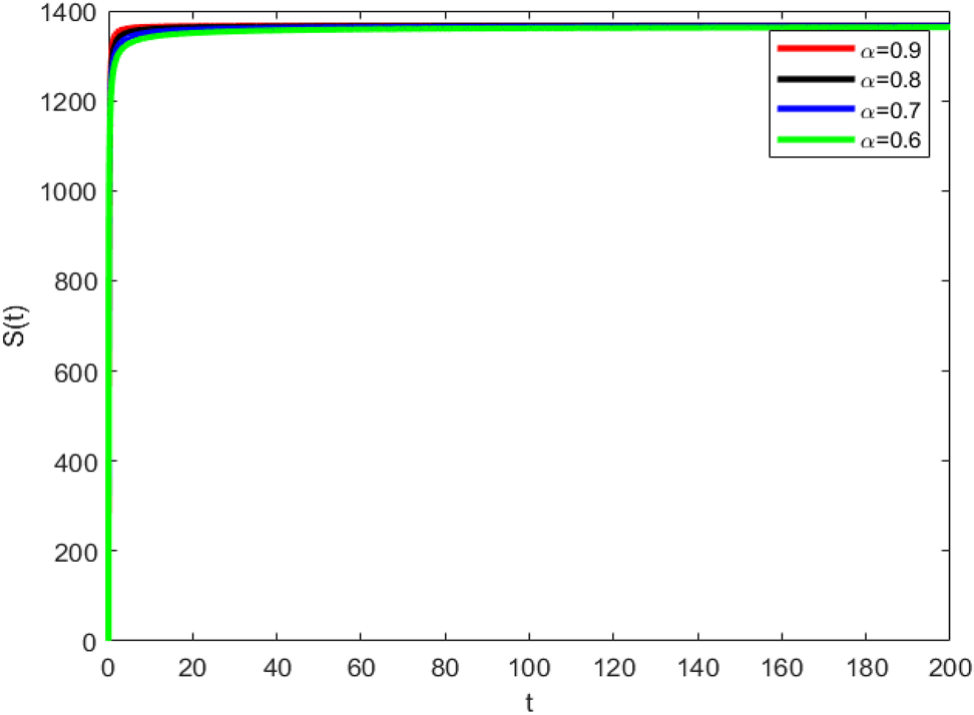
Graphical behavior of susceptible population (disease present in the population) for various values of fractional order $\alpha$.

This section is devoted for investigating the key properties of the simulated graphs against the set of parametric values as mentioned in Table 1. Further, these graphs are plotted when the disease is prevailing in the human population and attains the endemic steady state with in due course of time. To analyze the dynamics of the susceptible individuals at endemic equilibrium, four appropriate values of $\alpha$ are selected. We have practically noticed in the outburst of COVID-19 that it propagated with different rates in the different countries of the world. These rates are biologically meaning full as every individual or group of individuals have different physical environments, immunity levels, health conditions and leaving standards etc. In this regard, fractional parameter $\alpha$ handles the rate of spread for the corona virus. Figure 1 shows the flow map of coronavirus model. All the graphs in Fig. 2 depicts the dynamical behavior of the susceptible population. Every trajectory of the curved graph reflects that how the susceptible compartment attains the endemic steady state for different values of $\alpha$, with a particular set of parametric values including the crowding effect. It can be observed that each graph indicates the convergence towards the exact fixed point. Also, each graph possesses a specific trajectory and rate of convergence according to the fractional order parameter $\alpha$.

**Table 1:** Set of parametric values per day.

Symbols	Value/per day	Source
$\Lambda$	$\mu \times N(0)$	Estimated
$\mu$	$\frac{1}{67.7\times 365}$	^23^
$\gamma$	0.5000	Fitted
$\beta$	0.4000	Fitted
${\alpha }_{1}$	0.5465	Fitted
$\delta$	0.5000	Fitted
${\delta }_{1}$	0.1000	Fitted
${\delta }_{2}$	$5.32978$	Fitted
$\sigma$	$\ge 0$	Fitted

**Figure 3:**
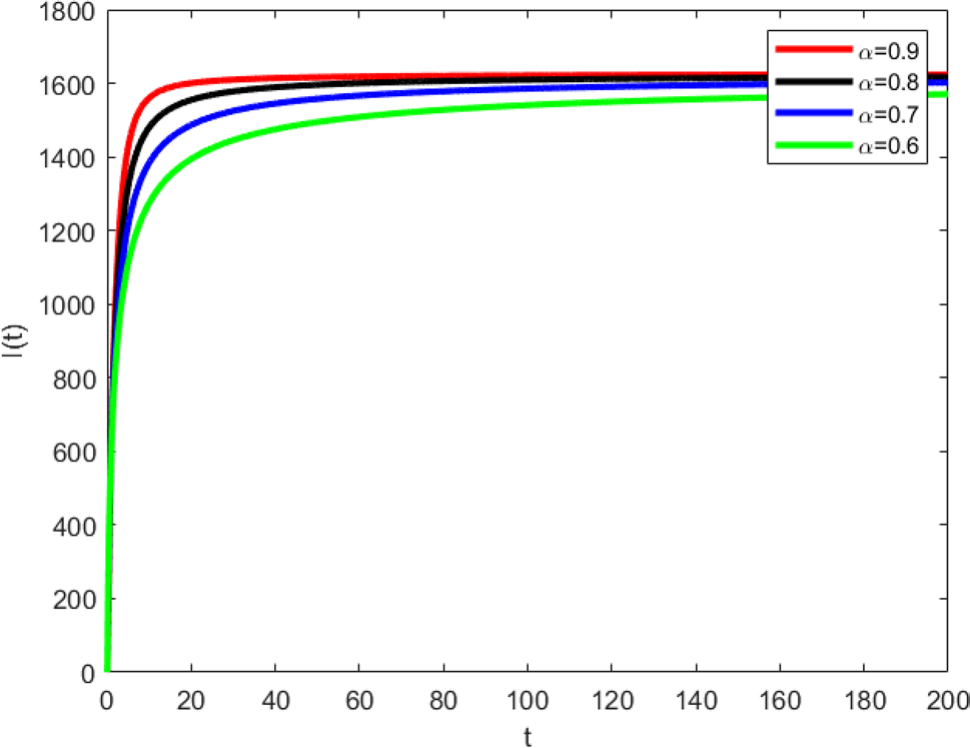
Graphical behavior of infected population (disease present in the population) for various values of fractional order $\alpha$.

Similarly, the curved graph in Fig. 3 shows the convergence of infected compartment, towards the exact fixed point. Infected graphs capture the dynamical evolution in the presence of viral load in the infected populace for the highlighted values of parameter. It is worth mentioning that each parameter has its own biological meaning and importance as described in the description of the model in section "Description of the model". All the graphs show the different phases of the disease dynamics according to the value of $\alpha$. If value of $\alpha$ is less then the rate of convergence is slow and vice versa. Moreover, the graphical behavior is in line with the real behavior of the continues system.

**Figure 4:**
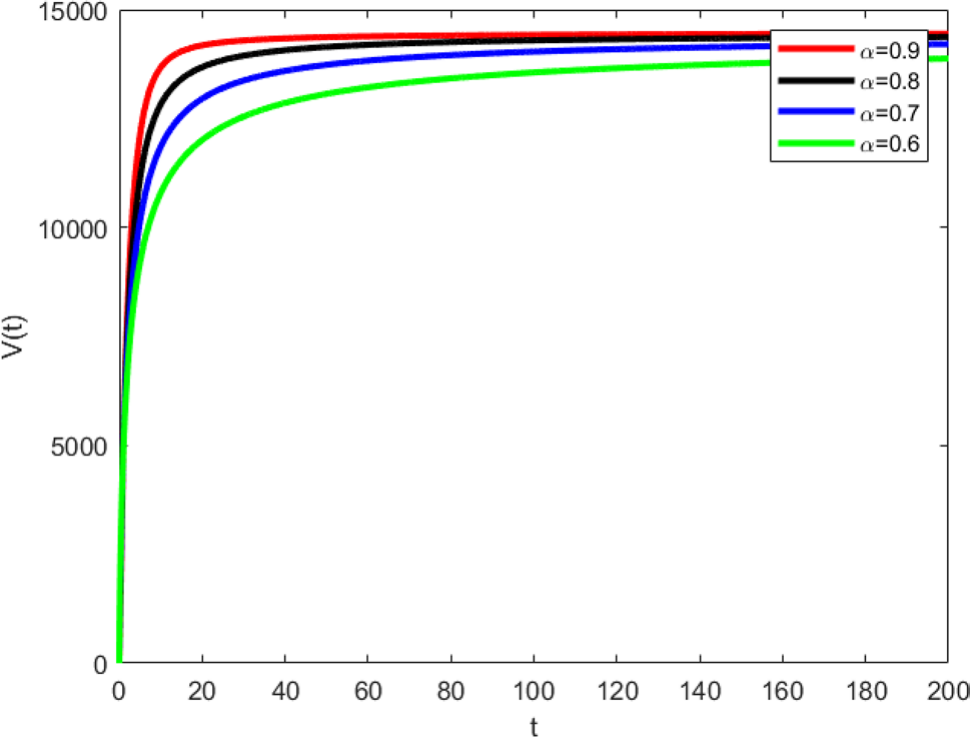
Graphical behavior of vaccinated population (disease present in the population) for various values of fractional order $\alpha$.

The set of the graphs in Fig. 4 demonstrate the vital behavior of vaccinated the populace in Covid-19 model. These graphs describe the evolutionary behavior of the vaccinated individuals during the propagation of Covid-19. Every graph has the specific trajectory and a different rate of convergence to attain the required steady state. Thus, fractional order parameter $\alpha$ plays an important role in describing the disease dynamics of every compartment.

**Figure 5:**
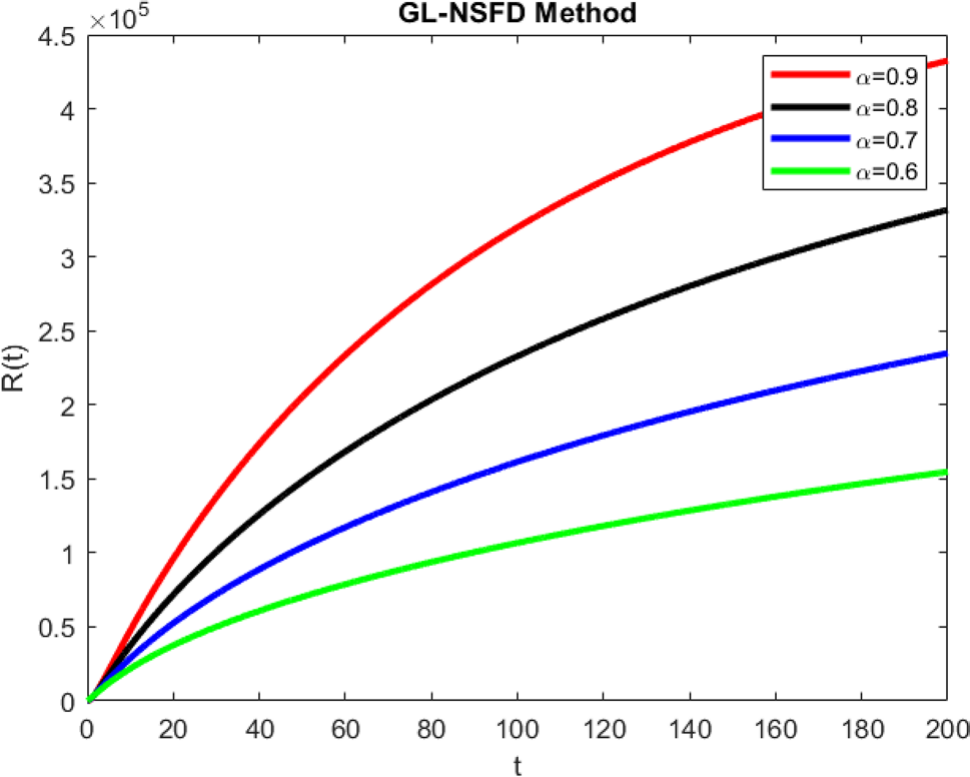
Graphical behavior of recovered population (disease present in the population) for various values of fractional order $\alpha$.

Finally, the graphs in Fig. 5 shows the dynamical behavior of the recovered population, when the Covid-19 disease persists in the community. The graphs bring an important fact into the lime light that the fast recovery rate can be captured by a large value of $\alpha$. Similarly, the slower rate of recovery may be represented by a smaller value of $\alpha .$

### Effect of vaccination

**Figure 6:**
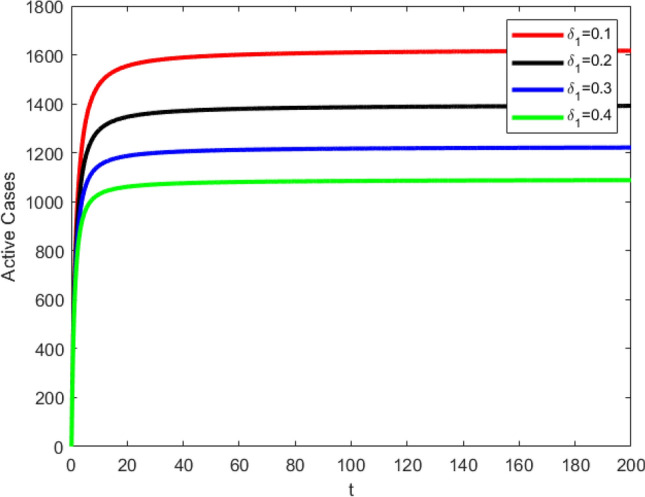
Effect of vaccination on active cases when fractional order value $\alpha =0.8$.

Vaccine of any disease plays a vital role in controlling the disease. Likewise, vaccination is an important strategy to control the Covid-19. The graphs in Fig. 6 depict the role of vaccinating for stopping the spread of the virus. All the graphs are drawn by choosing the $\alpha =0.8$ and values of vaccination parameter ${\delta }_{1}$ are shown in the values of vaccination. When the value of ${\delta }_{1}$ is less i.e. $0.1$, the number of infected individuals are greater as compared to the number of individuals against the ${\delta }_{1}=0.2, 0.3$ and $0.4.$

Consequently, the number of infected individuals $I\left(t\right)$ are inversely proportional to the vaccination parameters ${\delta }_{1}$. Hence, the parameter ${\delta }_{1}$ imparts a significant role to slow down the disease dynamics and the transmission of the virus can be controlled effectively by vaccinating a large part of population.

The main advantage of the theory, applied in this work is that it may be applied to examine the positivity, boundedness, stability analysis and uniquely existence of the solution for the fractional order epidemic models. On the other hand, if we look on the drawbacks or disadvantages of this study, one can notice that any epidemic model cannot be captured all the factors related to disease dynamics. So, there is a chance of error and slight deviation from the actual behavior of the disease.

## Numerical method

In this section, we construct nonstandard finite difference (NSFD) scheme using Grunwald–Letnikov approximation to numerically approximate Caputo fractional derivative^47–49^.

### Grunwald–Letnikov non-standard finite difference method

Let us now apply the Grunwald–Letnikov definition to the following fractional differential equation using the Caputo operator:

$${}_{0}{}^{c}{D}_{t}^{\alpha }y\left(t\right)={\text{f}}\left(y(t)\right) ,\mathrm{ y}\left(\mathcal{T}\right)={{\text{y}}}_{0} (0<\alpha <1),$$

And assume there exists a unique solution $y={\text{y}}\left(\mathcal{T}\right)$ in the interval $[0,\mathcal{T}]$ and let ${y}_{k}$ denote the approximation of the true solution $y({\mathcal{T}}_{{\text{k}}})$. Then the explicit or implicit Grunwald–Letnikov method for an equidistant grid is given by:

$${y}_{n+1}-\sum_{\nu =1}^{n+1}{C}_{\nu }^{\alpha }{y}_{n+1-\nu }-{r}_{n+1}^{\alpha }{y}_{0}={h}^{\alpha }f({y}_{n})$$ or $${h}^{\alpha }f({y}_{n+1}).$$

Where $${C}_{\nu }^{\alpha }=-{\left(1\right)}^{\nu -1}\left(\begin{array}{c}\alpha \\ \nu \end{array}\right) , {r}_{n+1}^{\alpha }={h}^{\alpha }{r}_{0}^{\alpha }\left({\mathcal{T}}_{{\text{n}}+1}\right)={\gamma }_{0,-1}^{\alpha }{\left(n+1\right)}^{-\alpha } ,$$

and the coefficients ${\gamma }_{0,-1}^{\alpha }=\frac{\Gamma (\mu \alpha +1)}{\Gamma (k\alpha +1)} , \mu ,k\in {\mathbb{N}}_{0}\cup \left\{-1\right\}.$

Moreover, the coefficients ${C}_{\nu }^{\alpha }$ and ${r}_{\nu }^{\alpha }$ satisfy the relations given in the next lemma.

#### Lemma 3

(^48^) Assume that $0<\alpha <1$, then all the coefficients ${C}_{\nu }^{\alpha }$ are positive and show the behaviour ${C}_{\nu }^{\alpha }=O(\frac{1}{{\nu }^{1+\alpha }})$ as $\nu \to \infty$. Further, the coefficients ${C}_{\nu }^{\alpha }$ and ${r}_{\nu }^{\alpha }$ satisfy for $\nu >1$ the properties:

$$0<{C}_{\nu +1}^{\alpha }<{C}_{\nu }^{\alpha }<\dots <{C}_{1}^{\alpha }=\alpha <1\mathrm{ and }0<{r}_{\nu +1}^{\alpha }<{r}_{\nu }^{\alpha }<\dots <{r}_{1}^{\alpha }=\frac{1}{\Gamma (1-\alpha )}.$$

#### Proof

It is clear that for all $\alpha \in \left(\mathrm{0,1}\right],$

$$\underset{\nu \to \infty }{{\text{lim}}}{C}_{\nu }^{\alpha }=0 , \underset{\nu \to \infty }{{\text{lim}}}{r}_{\nu }^{\alpha }=0.$$

Moreover, for $\alpha \in \left(\mathrm{0,1}\right],$ is $\alpha +\frac{1}{\Gamma (1-\alpha )}>1.$

It also shows that $\sum_{\nu =1}^{\infty }{C}_{\nu }^{\alpha }=1.$

Which is the result that will used later.

Now, we provide a non-standard finite-difference discretization of the mathematical model (1).

To this, we divide the interval $[0, L]$ into M ∈ N subintervals, respectively, and step sizes $h =\frac{L}{M}$. The approximate solutions $S, I, V {\text{and}} R$ of (1) will be denoted as ${S}^{n}, {I}^{n}, {V}^{n}, {R}^{n}$, respectively, for each $n=\mathrm{0,1}, \dots ,N.$ Under the rules, the discretization of a system (1) is presented in^43^.

First equation of system (1):

$${}_{0}{}^{c}{D}_{t}^{\alpha }S\left(t\right)=\Lambda -\frac{{\beta }^{\alpha }SI}{1+{\alpha }_{1}^{\alpha }I }-({\delta }_{2}^{\alpha }+{\mu }^{\alpha })S.$$

Use Grunwald–Letnikov approximation ${}_{0}{}^{c}{D}_{t}^{\alpha }y\left({t}_{n+1}\right)=\frac{1}{\psi {\left(h\right)}^{\alpha }}\left\{{y}_{n+1}-\sum_{\nu =1}^{n+1}{C}_{\nu }^{\alpha }{y}_{n+1-\nu }-{r}_{n+1}^{\alpha }{y}_{0}\right\}$ to left hand side of above equation, we have:

$$\frac{1}{\psi {\left(h\right)}^{\alpha }}\left\{{S}_{n+1}-\sum_{\nu =1}^{n+1}{C}_{\nu }^{\alpha }{S}_{n+1-\nu }-{r}_{n+1}^{\alpha }{S}_{0}\right\}=\Lambda -\frac{{\beta }^{\alpha }{S}_{n+1}{I}_{n}}{1+{\alpha }_{1}^{\alpha }{I}_{n} }-({\delta }_{2}^{\alpha }+{\mu }^{\alpha }){S}_{n+1},$$

$${S}_{n+1}-\sum_{\nu =1}^{n+1}{C}_{\nu }^{\alpha }{S}_{n+1-\nu }-{r}_{n+1}^{\alpha }{S}_{0}=\Lambda \psi {\left(h\right)}^{\alpha }-\psi {\left(h\right)}^{\alpha }\frac{{\beta }^{\alpha }{S}_{n+1}{I}_{n}}{1+{\alpha }_{1}^{\alpha }{I}_{n} }-\psi {\left(h\right)}^{\alpha }({\delta }_{2}^{\alpha }+{\mu }^{\alpha }){S}_{n+1}.$$

By shifting ${S}_{n+1}$ terms to L.H.S.

$${S}_{n+1}+\psi {\left(h\right)}^{\alpha }\frac{{\beta }^{\alpha }{S}_{n+1}{I}_{n}}{1+{\alpha }_{1}^{\alpha }{I}_{n} }+\psi {\left(h\right)}^{\alpha }\left({\delta }_{2}^{\alpha }+{\mu }^{\alpha }\right){S}_{n+1}=\Lambda \psi {\left(h\right)}^{\alpha }+{r}_{n+1}^{\alpha }{S}_{0}+\sum_{\nu =1}^{n+1}{C}_{\nu }^{\alpha }{S}_{n+1-\nu },$$

$${S}_{n+1}\left(1+\psi {\left(h\right)}^{\alpha }\frac{{\beta }^{\alpha }{I}_{n}}{1+{\alpha }_{1}^{\alpha }{I}_{n} }+\psi {\left(h\right)}^{\alpha }\left({\delta }_{2}^{\alpha }+{\mu }^{\alpha }\right)\right)=\Lambda \psi {\left(h\right)}^{\alpha }+{r}_{n+1}^{\alpha }{S}_{0}+\sum_{\nu =1}^{n+1}{C}_{\nu }^{\alpha }{S}_{n+1-\nu },$$

$${S}_{n+1}=\frac{\Lambda \psi {\left(h\right)}^{\alpha }+{r}_{n+1}^{\alpha }{S}_{0}+\sum_{\nu =1}^{n+1}{C}_{\nu }^{\alpha }{S}_{n+1-\nu }}{1+\psi {\left(h\right)}^{\alpha }\frac{{\beta }^{\alpha }{I}_{n}}{1+{\alpha }_{1}^{\alpha }{I}_{n} }+\psi {\left(h\right)}^{\alpha }\left({\delta }_{2}^{\alpha }+{\mu }^{\alpha }\right)}.$$

Second equation of system (1): ${}_{0}{}^{c}{D}_{t}^{\alpha }I\left(t\right)=\frac{{\beta }^{\alpha }SI}{1+{\alpha }_{1}^{\alpha }I }-({\gamma }^{\alpha }+{\delta }_{1}^{\alpha }+{\mu }^{\alpha })I.$

Use Grunwald–Letnikov approximation, ${}_{0}{}^{c}{D}_{t}^{\alpha }y\left({t}_{n+1}\right)=\frac{1}{\psi {\left(h\right)}^{\alpha }}\left\{{y}_{n+1}-\sum_{\nu =1}^{n+1}{C}_{\nu }^{\alpha }{y}_{n+1-\nu }-{r}_{n+1}^{\alpha }{y}_{0}\right\}$ to left hand side of above equation, we have:

$$\frac{1}{\psi {\left(h\right)}^{\alpha }}\left\{{I}_{n+1}-\sum_{\nu =1}^{n+1}{C}_{\nu }^{\alpha }{I}_{n+1-\nu }-{r}_{n+1}^{\alpha }{I}_{0}\right\}=\frac{{\beta }^{\alpha }{S}_{n+1}{I}_{n}}{1+{\alpha }_{1}^{\alpha }{I}_{n} }-({\gamma }^{\alpha }+{\delta }_{1}^{\alpha }+{\mu }^{\alpha }){I}_{n+1},$$

$${I}_{n+1}-\sum_{\nu =1}^{n+1}{C}_{\nu }^{\alpha }{I}_{n+1-\nu }-{r}_{n+1}^{\alpha }{I}_{0}=\psi {\left(h\right)}^{\alpha }\frac{{\beta }^{\alpha }{S}_{n+1}{I}_{n}}{1+{\alpha }_{1}^{\alpha }{I}_{n} }-\psi {\left(h\right)}^{\alpha }({\gamma }^{\alpha }+{\delta }_{1}^{\alpha }+{\mu }^{\alpha }){I}_{n+1}.$$

By shifting ${I}_{n+1}$ terms to L.H.S.

$${I}_{n+1}+\psi {\left(h\right)}^{\alpha }\left({\gamma }^{\alpha }+{\delta }_{1}^{\alpha }+{\mu }^{\alpha }\right){I}_{n+1}=\psi {\left(h\right)}^{\alpha }\frac{{\beta }^{\alpha }{S}_{n+1}{I}_{n}}{1+{\alpha }_{1}^{\alpha }{I}_{n} }{+r}_{n+1}^{\alpha }{I}_{0}+\sum_{\nu =1}^{n+1}{C}_{\nu }^{\alpha }{I}_{n+1-\nu },$$

$${I}_{n+1}\left(1++\psi {\left(h\right)}^{\alpha }\left({\gamma }^{\alpha }+{\delta }_{1}^{\alpha }+{\mu }^{\alpha }\right)\right)=\psi {\left(h\right)}^{\alpha }\frac{{\beta }^{\alpha }{S}_{n+1}{I}_{n}}{1+{\alpha }_{1}^{\alpha }{I}_{n} }+{r}_{n+1}^{\alpha }{I}_{0}+\sum_{\nu =1}^{n+1}{C}_{\nu }^{\alpha }{I}_{n+1-\nu ,}$$

$${I}_{n+1}=\frac{\psi {\left(h\right)}^{\alpha }\frac{{\beta }^{\alpha }{S}_{n+1}{I}_{n}}{1+{\alpha }_{1}^{\alpha }{I}_{n} }+{r}_{n+1}^{\alpha }{I}_{0}+\sum_{\nu =1}^{n+1}{C}_{\nu }^{\alpha }{I}_{n+1-\nu }}{1+\psi {\left(h\right)}^{\alpha }\left({\gamma }^{\alpha }+{\delta }_{1}^{\alpha }+{\mu }^{\alpha }\right)}.$$

Third equation of system (1): ${}_{0}{}^{c}{D}_{t}^{\alpha }V\left(t\right)={\delta }_{2}^{\alpha }S+{\delta }_{1}^{\alpha }I-({\sigma }^{\alpha }+{\mu }^{\alpha })V.$

Use Grunwald–Letnikov approximation ${}_{0}{}^{c}{D}_{t}^{\alpha }y\left({t}_{n+1}\right)=\frac{1}{\psi {\left(h\right)}^{\alpha }}\left\{{y}_{n+1}-\sum_{\nu =1}^{n+1}{C}_{\nu }^{\alpha }{y}_{n+1-\nu }-{r}_{n+1}^{\alpha }{y}_{0}\right\}$ to left hand side of above equation, we have:

$$\frac{1}{\psi {\left(h\right)}^{\alpha }}\left\{{V}_{n+1}-\sum_{\nu =1}^{n+1}{C}_{\nu }^{\alpha }{V}_{n+1-\nu }-{r}_{n+1}^{\alpha }{V}_{0}\right\}={\delta }_{2}^{\alpha }{S}_{n+1}+{\delta }_{1}^{\alpha }{I}_{n+1}-\left({\sigma }^{\alpha }+{\mu }^{\alpha }\right){V}^{n+1},$$

$${V}_{n+1}-\sum_{\nu =1}^{n+1}{C}_{\nu }^{\alpha }{V}_{n+1-\nu }-{r}_{n+1}^{\alpha }{V}_{0}={\psi {\left(h\right)}^{\alpha }\delta }_{2}^{\alpha }{S}_{n+1}+{\psi {\left(h\right)}^{\alpha }\delta }_{1}^{\alpha }{I}_{n+1}-\psi {\left(h\right)}^{\alpha }\left({\sigma }^{\alpha }+{\mu }^{\alpha }\right){V}^{n+1},$$

$${V}_{n+1}+\psi {\left(h\right)}^{\alpha }\left({\sigma }^{\alpha }+{\mu }^{\alpha }\right){V}^{n+1}=\sum_{\nu =1}^{n+1}{C}_{\nu }^{\alpha }{V}_{n+1-\nu }+{\psi {\left(h\right)}^{\alpha }\delta }_{2}^{\alpha }{S}_{n+1}+{\psi {\left(h\right)}^{\alpha }\delta }_{1}^{\alpha }{I}_{n+1}+{r}_{n+1}^{\alpha }{V}_{0},$$

$${V}_{n+1}\left(1+\psi {\left(h\right)}^{\alpha }\left({\sigma }^{\alpha }+{\mu }^{\alpha }\right)\right)=\sum_{\nu =1}^{n+1}{C}_{\nu }^{\alpha }{V}_{n+1-\nu }+{\psi {\left(h\right)}^{\alpha }\delta }_{2}^{\alpha }{S}_{n+1}+{\psi {\left(h\right)}^{\alpha }\delta }_{1}^{\alpha }{I}_{n+1}+{r}_{n+1}^{\alpha }{V}_{0},$$

$${V}_{n+1}=\frac{{\sum_{\nu =1}^{n+1}{C}_{\nu }^{\alpha }{V}_{n+1-\nu }+\psi {\left(h\right)}^{\alpha }\delta }_{2}^{\alpha }{S}_{n+1}+{\psi {\left(h\right)}^{\alpha }\delta }_{1}^{\alpha }{I}_{n+1}+{r}_{n+1}^{\alpha }{V}_{0}}{1+\psi {\left(h\right)}^{\alpha }\left({\sigma }^{\alpha }+{\mu }^{\alpha }\right)}.$$

Fourth equation of system (1):

$${}_{0}{}^{c}{D}_{t}^{\alpha }R\left(t\right)={\gamma }^{\alpha }I+{\sigma }^{\alpha }V-{\mu }^{\alpha }R.$$

Use Grunwald–Letnikov approximation ${}_{0}{}^{c}{D}_{t}^{\alpha }y\left({t}_{n+1}\right)=\frac{1}{\psi {\left(h\right)}^{\alpha }}\left\{{y}_{n+1}-\sum_{\nu =1}^{n+1}{C}_{\nu }^{\alpha }{y}_{n+1-\nu }-{r}_{n+1}^{\alpha }{y}_{0}\right\}$ to left hand side of above equation, we have:

$$\frac{1}{\psi {\left(h\right)}^{\alpha }}\left\{{R}_{n+1}-\sum_{\nu =1}^{n+1}{C}_{\nu }^{\alpha }{R}_{n+1-\nu }-{r}_{n+1}^{\alpha }{R}_{0}\right\}={\gamma }^{\alpha }{I}_{n+1}+{\sigma }^{\alpha }{V}_{n+1}-{\mu }^{\alpha }{R}_{n+1},$$

$${R}_{n+1}-\sum_{\nu =1}^{n+1}{C}_{\nu }^{\alpha }{R}_{n+1-\nu }-{r}_{n+1}^{\alpha }{R}_{0}=\psi {\left(h\right)}^{\alpha }{\gamma }^{\alpha }{I}_{n+1}+\psi {\left(h\right)}^{\alpha }{\sigma }^{\alpha }{V}_{n+1}-\psi {\left(h\right)}^{\alpha }{\mu }^{\alpha }{R}_{n+1},$$

$${R}_{n+1}+\psi {\left(h\right)}^{\alpha }{\mu }^{\alpha }{R}_{n+1}=\sum_{\nu =1}^{n+1}{C}_{\nu }^{\alpha }{R}_{n+1-\nu }+{r}_{n+1}^{\alpha }{R}_{0}+\psi {\left(h\right)}^{\alpha }{\gamma }^{\alpha }{I}_{n+1}+\psi {\left(h\right)}^{\alpha }{\sigma }^{\alpha }{V}_{n+1},$$

$${R}_{n+1}\left(1+\psi {\left(h\right)}^{\alpha }{\mu }^{\alpha }\right)=\sum_{\nu =1}^{n+1}{C}_{\nu }^{\alpha }{R}_{n+1-\nu }+{r}_{n+1}^{\alpha }{R}_{0}+\psi {\left(h\right)}^{\alpha }{\gamma }^{\alpha }{I}_{n+1}+\psi {\left(h\right)}^{\alpha }{\sigma }^{\alpha }{V}_{n+1},$$

$${R}_{n+1}=\frac{\sum_{\nu =1}^{n+1}{C}_{\nu }^{\alpha }{R}_{n+1-\nu }+{r}_{n+1}^{\alpha }{R}_{0}+\psi {\left(h\right)}^{\alpha }{\gamma }^{\alpha }{I}_{n+1}+\psi {\left(h\right)}^{\alpha }{\sigma }^{\alpha }{V}_{n+1}}{1+\psi {\left(h\right)}^{\alpha }{\mu }^{\alpha }}.$$

Now we have a system of equations:

$${S}_{n+1}=\frac{\Lambda \psi {\left(h\right)}^{\alpha }+{r}_{n+1}^{\alpha }{S}_{0}+\sum_{\nu =1}^{n+1}{C}_{\nu }^{\alpha }{S}_{n+1-\nu }}{1+\psi {\left(h\right)}^{\alpha }\frac{{\beta }^{\alpha }{I}_{n}}{1+{\alpha }_{1}^{\alpha }{I}_{n} }+\psi {\left(h\right)}^{\alpha }\left({\delta }_{2}^{\alpha }+{\mu }^{\alpha }\right)},$$

$${I}_{n+1}=\frac{\psi {\left(h\right)}^{\alpha }\frac{{\beta }^{\alpha }{S}_{n+1}{I}_{n}}{1+{\alpha }_{1}^{\alpha }{I}_{n} }+{r}_{n+1}^{\alpha }{I}_{0}+\sum_{\nu =1}^{n+1}{C}_{\nu }^{\alpha }{I}_{n+1-\nu }}{1+\psi {\left(h\right)}^{\alpha }\left({\gamma }^{\alpha }+{\delta }_{1}^{\alpha }+{\mu }^{\alpha }\right)},$$

$${V}_{n+1}=\frac{{\sum_{\nu =1}^{n+1}{C}_{\nu }^{\alpha }{V}_{n+1-\nu }+\psi {\left(h\right)}^{\alpha }\delta }_{2}^{\alpha }{S}_{n+1}+{\psi {\left(h\right)}^{\alpha }\delta }_{1}^{\alpha }{I}_{n+1}+{r}_{n+1}^{\alpha }{V}_{0}}{1+\psi {\left(h\right)}^{\alpha }\left({\sigma }^{\alpha }+{\mu }^{\alpha }\right)},$$

$${R}_{n+1}=\frac{\sum_{\nu =1}^{n+1}{C}_{\nu }^{\alpha }{R}_{n+1-\nu }+{r}_{n+1}^{\alpha }{R}_{0}+\psi {\left(h\right)}^{\alpha }{\gamma }^{\alpha }{I}_{n+1}+\psi {\left(h\right)}^{\alpha }{\sigma }^{\alpha }{V}_{n+1}}{1+\psi {\left(h\right)}^{\alpha }{\mu }^{\alpha }}.$$

Next, we establish the most important properties of NSFD method^48^.

### Properties

#### Theorem 8

(Positivity$$)$$ The deterministic form of SIVR model system preserves the non-negativity of the solution.

#### Proof

We will prove this by induction method.

Suppose that ${S}_{0}\ge 0, {I}_{0}\ge 0, {V}_{0}\ge 0, {R}_{0}\ge 0$, then ${S}_{n}\ge 0, {I}_{n}\ge 0, {V}_{n}\ge 0, {R}_{n}\ge 0$ is satisfied for $n=\mathrm{1,2},\dots$

By induction, for $n=0$, above system can be written as:

$${S}_{1}=\frac{\Lambda \psi {\left(h\right)}^{\alpha }+{r}_{1}^{\alpha }{S}_{0}+{C}_{1}^{\alpha }{S}_{0}}{1+\psi {\left(h\right)}^{\alpha }\frac{{\beta }^{\alpha }{I}_{0}}{1+{\alpha }_{1}^{\alpha }{I}_{0} }+\psi {\left(h\right)}^{\alpha }\left({\delta }_{2}^{\alpha }+{\mu }^{\alpha }\right)}\ge 0,$$

$${I}_{1}=\frac{\psi {\left(h\right)}^{\alpha }\frac{{\beta }^{\alpha }{S}_{1}{I}_{0}}{1+{\alpha }_{1}^{\alpha }{I}_{0} }+{r}_{1}^{\alpha }{I}_{0}+{C}_{1}^{\alpha }{I}_{0}}{1+\psi {\left(h\right)}^{\alpha }\left({\gamma }^{\alpha }+{\delta }_{1}^{\alpha }+{\mu }^{\alpha }\right)}\ge 0,$$

$${V}_{1}=\frac{{{C}_{1}^{\alpha }{V}_{0}+\psi {\left(h\right)}^{\alpha }\delta }_{2}^{\alpha }{S}_{1}+{\psi {\left(h\right)}^{\alpha }\delta }_{1}^{\alpha }{I}_{1}+{r}_{1}^{\alpha }{V}_{0}}{1+\psi {\left(h\right)}^{\alpha }\left({\sigma }^{\alpha }+{\mu }^{\alpha }\right)}\ge 0,$$

$${R}_{1}=\frac{{C}_{1}^{\alpha }{R}_{0}+{r}_{1}^{\alpha }{R}_{0}+\psi {\left(h\right)}^{\alpha }{\gamma }^{\alpha }{I}_{1}+\psi {\left(h\right)}^{\alpha }{\sigma }^{\alpha }{V}_{1}}{1+\psi {\left(h\right)}^{\alpha }{\mu }^{\alpha }}\ge 0.$$

As all the parameters are positive, so that, ${S}_{1}\ge 0, {I}_{1}\ge 0, {V}_{1}\ge 0, {R}_{1}\ge 0.$

Now, we will suppose that for positive integers $\mathrm{1,2}, \dots , n-1, {S}_{n}\ge 0, {I}_{n}\ge 0, {V}_{n}\ge 0, {R}_{n}\ge 0$, i.e. ${S}_{2}, {S}_{3}, \dots ,{S}_{n}\ge 0, {I}_{2}, {I}_{3},\dots .,{I}_{n}\ge 0, {V}_{1}, {V}_{2}, \dots .,{V}_{n}\ge 0, {R}_{1},{R}_{2},\dots ,{R}_{n}\ge 0.$

$${S}_{n}=\frac{\Lambda \psi {\left(h\right)}^{\alpha }+{r}_{n}^{\alpha }{S}_{0}+\sum_{\nu =1}^{n}{C}_{\nu }^{\alpha }{S}_{n-\nu }}{1+\psi {\left(h\right)}^{\alpha }\frac{{\beta }^{\alpha }{I}_{n-1}}{1+{\alpha }_{1}^{\alpha }{I}_{n-1} }+\psi {\left(h\right)}^{\alpha }\left({\delta }_{2}^{\alpha }+{\mu }^{\alpha }\right)}\ge 0, \forall n\in \left\{\mathrm{1,2},3,\dots n-1\right\},$$

$${I}_{n}=\frac{\psi {\left(h\right)}^{\alpha }\frac{{\beta }^{\alpha }{S}_{n}{I}_{n-1}}{1+{\alpha }_{1}^{\alpha }{I}_{n-1} }+{r}_{n}^{\alpha }{I}_{0}+\sum_{\nu =1}^{n}{C}_{\nu }^{\alpha }{I}_{n-\nu }}{1+\psi {\left(h\right)}^{\alpha }\left({\gamma }^{\alpha }+{\delta }_{1}^{\alpha }+{\mu }^{\alpha }\right)}\ge 0 , \forall n\in \left\{\mathrm{1,2},3,\dots n-1\right\},$$

$${V}_{n}=\frac{{\sum_{\nu =1}^{n}{C}_{\nu }^{\alpha }{V}_{n-\nu }+\psi {\left(h\right)}^{\alpha }\delta }_{2}^{\alpha }{S}_{n}+{\psi {\left(h\right)}^{\alpha }\delta }_{1}^{\alpha }{I}_{n}+{r}_{n}^{\alpha }{V}_{0}}{1+\psi {\left(h\right)}^{\alpha }\left({\sigma }^{\alpha }+{\mu }^{\alpha }\right)}\ge 0, \forall n\in \left\{\mathrm{1,2},3,\dots n-1\right\},$$

$${R}_{n}=\frac{\sum_{\nu =1}^{n}{C}_{\nu }^{\alpha }{R}_{n-\nu }+{r}_{n}^{\alpha }{R}_{0}+\psi {\left(h\right)}^{\alpha }{\gamma }^{\alpha }{I}_{n}+\psi {\left(h\right)}^{\alpha }{\sigma }^{\alpha }{V}_{n}}{1+\psi {\left(h\right)}^{\alpha }{\mu }^{\alpha }}\ge 0, \forall n\in \left\{\mathrm{1,2},3,\dots n-1\right\}.$$

By using above results, for positive integer $n$ i.e. $\in {\mathbb{Z}}^{+}$ , we conclude that:

$${S}_{n+1}=\frac{\Lambda \psi {\left(h\right)}^{\alpha }+{r}_{n+1}^{\alpha }{S}_{0}+\sum_{\nu =1}^{n+1}{C}_{\nu }^{\alpha }{S}_{n+1-\nu }}{1+\psi {\left(h\right)}^{\alpha }\frac{{\beta }^{\alpha }{I}_{n}}{1+{\alpha }_{1}^{\alpha }{I}_{n} }+\psi {\left(h\right)}^{\alpha }\left({\delta }_{2}^{\alpha }+{\mu }^{\alpha }\right)}\ge 0,$$

$${I}_{n+1}=\frac{\psi {\left(h\right)}^{\alpha }\frac{{\beta }^{\alpha }{S}_{n+1}{I}_{n}}{1+{\alpha }_{1}^{\alpha }{I}_{n} }+{r}_{n+1}^{\alpha }{I}_{0}+\sum_{\nu =1}^{n+1}{C}_{\nu }^{\alpha }{I}_{n+1-\nu }}{1+\psi {\left(h\right)}^{\alpha }\left({\gamma }^{\alpha }+{\delta }_{1}^{\alpha }+{\mu }^{\alpha }\right)}\ge 0,$$

$${V}_{n+1}=\frac{{\sum_{\nu =1}^{n+1}{C}_{\nu }^{\alpha }{V}_{n+1-\nu }+\psi {\left(h\right)}^{\alpha }\delta }_{2}^{\alpha }{S}_{n+1}+{\psi {\left(h\right)}^{\alpha }\delta }_{1}^{\alpha }{I}_{n+1}+{r}_{n+1}^{\alpha }{V}_{0}}{1+\psi {\left(h\right)}^{\alpha }\left({\sigma }^{\alpha }+{\mu }^{\alpha }\right)}\ge 0,$$

$${R}_{n+1}=\frac{\sum_{\nu =1}^{n+1}{C}_{\nu }^{\alpha }{R}_{n+1-\nu }+{r}_{n+1}^{\alpha }{R}_{0}+\psi {\left(h\right)}^{\alpha }{\gamma }^{\alpha }{I}_{n+1}+\psi {\left(h\right)}^{\alpha }{\sigma }^{\alpha }{V}_{n+1}}{1+\psi {\left(h\right)}^{\alpha }{\mu }^{\alpha }}\ge 0.$$

Hence all the state variables in the discretized model guarantee the positivity of the solution, as desired.

#### Theorem 9

(Boundedness) Suppose that ${\alpha }_{1}^{\alpha }>0, {{\delta }_{1}^{\alpha }>0, \delta }_{2}^{\alpha }>0, {\mu }^{\alpha }>0, {\gamma }^{\alpha }>0, {\sigma }^{\alpha }>0, \psi {\left(h\right)}^{\alpha }>0$ For all $\alpha \in (\mathrm{0,1})$, then there is a constant:

$$N\left({N}_{0}+1,\alpha \right)=\frac{N({N}_{0},\alpha )+\frac{1}{\Gamma (1-\mathrm{\alpha })}+\psi {\left(h\right)}^{\alpha }\Lambda }{1+\psi {\left(h\right)}^{\alpha }{\mu }^{\alpha }}.$$

Such that ${S}_{n+1}, {I}_{n+1}, {I}_{n+1}, {R}_{n+1}\le N\left({N}_{0}+1,\alpha \right)$ for $n=0, 1, 2, \dots ,{N}_{0}.$

#### Proof

$${S}_{n+1}=\frac{\Lambda \psi {\left(h\right)}^{\alpha }+{r}_{n+1}^{\alpha }{S}_{0}+\sum_{\nu =1}^{n+1}{C}_{\nu }^{\alpha }{S}_{n+1-\nu }}{1+\psi {\left(h\right)}^{\alpha }\frac{{\beta }^{\alpha }{I}_{n}}{1+{\alpha }_{1}^{\alpha }{I}_{n} }+\psi {\left(h\right)}^{\alpha }\left({\delta }_{2}^{\alpha }+{\mu }^{\alpha }\right)},$$

$${S}_{n+1}\left(1+\psi {\left(h\right)}^{\alpha }\frac{{\beta }^{\alpha }{I}_{n}}{1+{\alpha }_{1}^{\alpha }{I}_{n} }+\psi {\left(h\right)}^{\alpha }\left({\delta }_{2}^{\alpha }+{\mu }^{\alpha }\right)\right)=\Lambda \psi {\left(h\right)}^{\alpha }+{r}_{n+1}^{\alpha }{S}_{0}+\sum_{\nu =1}^{n+1}{C}_{\nu }^{\alpha }{S}_{n+1-\nu ,}$$

$${S}_{n+1}\left(1+\psi {\left(h\right)}^{\alpha }\frac{{\beta }^{\alpha }{I}_{n}}{1+{\alpha }_{1}^{\alpha }{I}_{n} }+\psi {\left(h\right)}^{\alpha }\left({\delta }_{2}^{\alpha }+{\mu }^{\alpha }\right)\right)=\Lambda \psi {\left(h\right)}^{\alpha }+{r}_{n+1}^{\alpha }{S}_{0}+\sum_{\nu =1}^{n+1}{C}_{\nu }^{\alpha }{S}_{n+1-\nu },$$

$${S}_{n+1}+\psi {\left(h\right)}^{\alpha }\frac{{\beta }^{\alpha }{I}_{n}}{1+{\alpha }_{1}^{\alpha }{I}_{n} }{S}_{n+1}+\psi {\left(h\right)}^{\alpha }{\delta }_{2}^{\alpha }{S}_{n+1}++\psi {\left(h\right)}^{\alpha }{\mu }^{\alpha }{S}_{n+1}=\Lambda \psi {\left(h\right)}^{\alpha }+{r}_{n+1}^{\alpha }{S}_{0}+\sum_{\nu =1}^{n+1}{C}_{\nu }^{\alpha }{S}_{n+1-\nu .}$$

$${I}_{n+1}=\frac{\psi {\left(h\right)}^{\alpha }\frac{{\beta }^{\alpha }{S}_{n+1}{I}_{n}}{1+{\alpha }_{1}^{\alpha }{I}_{n} }+{r}_{n+1}^{\alpha }{I}_{0}+\sum_{\nu =1}^{n+1}{C}_{\nu }^{\alpha }{I}_{n+1-\nu }}{1+\psi {\left(h\right)}^{\alpha }\left({\gamma }^{\alpha }+{\delta }_{1}^{\alpha }+{\mu }^{\alpha }\right)},$$

$${I}_{n+1}\left(1+\psi {\left(h\right)}^{\alpha }\left({\gamma }^{\alpha }+{\delta }_{1}^{\alpha }+{\mu }^{\alpha }\right)\right)=\psi {\left(h\right)}^{\alpha }\frac{{\beta }^{\alpha }{S}_{n+1}{I}_{n}}{1+{\alpha }_{1}^{\alpha }{I}_{n} }+{r}_{n+1}^{\alpha }{I}_{0}+\sum_{\nu =1}^{n+1}{C}_{\nu }^{\alpha }{I}_{n+1-\nu },$$

$${I}_{n+1}+\psi {\left(h\right)}^{\alpha }{\gamma }^{\alpha }{I}_{n+1}+\psi {\left(h\right)}^{\alpha }{{\delta }_{1}^{\alpha }I}_{n+1}+{\psi {\left(h\right)}^{\alpha }{\mu }^{\alpha }I}_{n+1}=\psi {\left(h\right)}^{\alpha }\frac{{\beta }^{\alpha }{S}_{n+1}{I}_{n}}{1+{\alpha }_{1}^{\alpha }{I}_{n} }+{r}_{n+1}^{\alpha }{I}_{0}+\sum_{\nu =1}^{n+1}{C}_{\nu }^{\alpha }{I}_{n+1-\nu },$$=

$${V}_{n+1}=\frac{{\sum_{\nu =1}^{n+1}{C}_{\nu }^{\alpha }{V}_{n+1-\nu }+\psi {\left(h\right)}^{\alpha }\delta }_{2}^{\alpha }{S}_{n+1}+{\psi {\left(h\right)}^{\alpha }\delta }_{1}^{\alpha }{I}_{n+1}+{r}_{n+1}^{\alpha }{V}_{0}}{1+\psi {\left(h\right)}^{\alpha }\left({\sigma }^{\alpha }+{\mu }^{\alpha }\right)},$$

$${V}_{n+1}\left(1+\psi {\left(h\right)}^{\alpha }\left({\sigma }^{\alpha }+{\mu }^{\alpha }\right)\right)={\sum_{\nu =1}^{n+1}{C}_{\nu }^{\alpha }{V}_{n+1-\nu }+\psi {\left(h\right)}^{\alpha }\delta }_{2}^{\alpha }{S}_{n+1}+{\psi {\left(h\right)}^{\alpha }\delta }_{1}^{\alpha }{I}_{n+1}+{r}_{n+1}^{\alpha }{V}_{0},$$

$${V}_{n+1}+\psi {\left(h\right)}^{\alpha }{\sigma }^{\alpha }{V}_{n+1}+{\psi {\left(h\right)}^{\alpha }{\mu }^{\alpha }V}_{n+1}={\sum_{\nu =1}^{n+1}{C}_{\nu }^{\alpha }{V}_{n+1-\nu }+\psi {\left(h\right)}^{\alpha }\delta }_{2}^{\alpha }{S}_{n+1}+{\psi {\left(h\right)}^{\alpha }\delta }_{1}^{\alpha }{I}_{n+1}+{r}_{n+1}^{\alpha }{V}_{0}.$$

$${R}_{n+1}=\frac{\sum_{\nu =1}^{n+1}{C}_{\nu }^{\alpha }{R}_{n+1-\nu }+{r}_{n+1}^{\alpha }{R}_{0}+\psi {\left(h\right)}^{\alpha }{\gamma }^{\alpha }{I}_{n+1}+\psi {\left(h\right)}^{\alpha }{\sigma }^{\alpha }{V}_{n+1}}{1+\psi {\left(h\right)}^{\alpha }{\mu }^{\alpha }},$$

$${R}_{n+1}\left(1+\psi {\left(h\right)}^{\alpha }{\mu }^{\alpha }\right)=\sum_{\nu =1}^{n+1}{C}_{\nu }^{\alpha }{R}_{n+1-\nu }+{r}_{n+1}^{\alpha }{R}_{0}+\psi {\left(h\right)}^{\alpha }{\gamma }^{\alpha }{I}_{n+1}+\psi {\left(h\right)}^{\alpha }{\sigma }^{\alpha }{V}_{n+1},$$

$${R}_{n+1}+\psi {\left(h\right)}^{\alpha }{\mu }^{\alpha }{R}_{n+1}=\sum_{\nu =1}^{n+1}{C}_{\nu }^{\alpha }{R}_{n+1-\nu }+{r}_{n+1}^{\alpha }{R}_{0}+\psi {\left(h\right)}^{\alpha }{\gamma }^{\alpha }{I}_{n+1}+\psi {\left(h\right)}^{\alpha }{\sigma }^{\alpha }{V}_{n+1}.$$

By adding the above equations, we have:

$$\begin{aligned} & S_{n + 1} + \psi \left( h \right)^{\alpha } \frac{{\beta^{\alpha } I_{n} }}{{1 + \alpha_{1}^{\alpha } I_{n} }}S_{n + 1} + \psi \left( h \right)^{\alpha } \delta_{2}^{\alpha } S_{n + 1} + + \psi \left( h \right)^{\alpha } \mu^{\alpha } S_{n + 1} + I_{n + 1} + \psi \left( h \right)^{\alpha } \gamma^{\alpha } I_{n + 1} \\ & \quad + \psi \left( h \right)^{\alpha } \delta_{1}^{\alpha } I_{n + 1} + \psi \left( h \right)^{\alpha } \mu^{\alpha } I_{n + 1} V_{n + 1} + \psi \left( h \right)^{\alpha } \sigma^{\alpha } V_{n + 1} + \psi \left( h \right)^{\alpha } \mu^{\alpha } V_{n + 1} + R_{n + 1} \\ & \quad + \psi \left( h \right)^{\alpha } \mu^{\alpha } R_{n + 1} \\ & = {\Lambda }\psi \left( h \right)^{\alpha } + r_{n + 1}^{\alpha } S_{0} + \mathop \sum \limits_{\nu = 1}^{n + 1} C_{\nu }^{\alpha } S_{n + 1 - \nu } + \psi \left( h \right)^{\alpha } \frac{{\beta^{\alpha } S_{n + 1} I_{n} }}{{1 + \alpha_{1}^{\alpha } I_{n} }} + r_{n + 1}^{\alpha } I_{0} \\ & \quad + \mathop \sum \limits_{\nu = 1}^{n + 1} C_{\nu }^{\alpha } I_{n + 1 - \nu } + \mathop \sum \limits_{\nu = 1}^{n + 1} C_{\nu }^{\alpha } V_{n + 1 - \nu } + \psi \left( h \right)^{\alpha } \delta_{2}^{\alpha } S_{n + 1} + \psi \left( h \right)^{\alpha } \delta_{1}^{\alpha } I_{n + 1} + r_{n + 1}^{\alpha } V_{0} \\ & \quad + \mathop \sum \limits_{\nu = 1}^{n + 1} C_{\nu }^{\alpha } R_{n + 1 - \nu } + r_{n + 1}^{\alpha } R_{0} + \psi \left( h \right)^{\alpha } \gamma^{\alpha } I_{n + 1} + \psi \left( h \right)^{\alpha } \sigma^{\alpha } V_{n + 1} . \\ \end{aligned}$$

$$\begin{aligned} & S_{n + 1} \left( {1 + \psi \left( h \right)^{\alpha } \mu^{\alpha } } \right) + I_{n + 1} \left( {1 + \psi \left( h \right)^{\alpha } \mu^{\alpha } } \right) + V_{n + 1} + R_{n + 1 } \left( {1 + \psi \left( h \right)^{\alpha } \mu^{\alpha } } \right) = {\Lambda }\psi \left( h \right)^{\alpha } \\ & + \mathop \sum \limits_{\nu = 1}^{n + 1} C_{\nu }^{\alpha } \left( {S_{n + 1 - \nu } + I_{n + 1 - \nu } + V_{n + 1 - \nu } + R_{n + 1 - \nu } } \right) + r_{n + 1}^{\alpha } \left( {S_{0} + I_{0} + V_{0} + R_{0} } \right), \\ \end{aligned}$$

$$\begin{aligned} & \left( {1 + \psi \left( h \right)^{\alpha } \mu^{\alpha } } \right)\left( {S_{n + 1} + I_{n + 1} + V_{n + 1} + R_{n + 1} } \right) = {\Lambda }\psi \left( h \right)^{\alpha } \\ & + \mathop \sum \limits_{\nu = 1}^{n + 1} C_{\nu }^{\alpha } \left( {S_{n + 1 - \nu } + I_{n + 1 - \nu } + V_{n + 1 - \nu } + R_{n + 1 - \nu } } \right) + r_{n + 1}^{\alpha } \left( {S_{0} + I_{0} + V_{0} + R_{0} } \right), \\ \end{aligned}$$

$$\begin{aligned} & S_{n + 1} + I_{n + 1} + V_{n + 1} + R_{n + 1} \\ & = \frac{{{\Lambda }\psi \left( h \right)^{\alpha } + \mathop \sum \nolimits_{\nu = 1}^{n + 1} C_{\nu }^{\alpha } \left( {S_{n + 1 - \nu } + I_{n + 1 - \nu } + V_{n + 1 - \nu } + R_{n + 1 - \nu } } \right) + r_{n + 1}^{\alpha } \left( {S_{0} + I_{0} + V_{0} + R_{0} } \right)}}{{1 + \psi \left( h \right)^{\alpha } \mu^{\alpha } }}, \\ \end{aligned}$$

$$\begin{aligned} & S_{n + 1} + I_{n + 1} + V_{n + 1} + R_{n + 1} \\ & = \frac{{{\Lambda }\psi \left( h \right)^{\alpha } + \mathop \sum \nolimits_{\nu = 1}^{n + 1} C_{\nu }^{\alpha } \left( {S_{n + 1 - \nu } + I_{n + 1 - \nu } + V_{n + 1 - \nu } + R_{n + 1 - \nu } } \right) + r_{n + 1}^{\alpha } }}{{1 + \psi \left( h \right)^{\alpha } \mu^{\alpha } }}, \\ \end{aligned}$$

For, $$n=0,$$

$${S}_{1}+{I}_{1}+{V}_{1}+{R}_{1}=\frac{\Lambda \psi {\left(h\right)}^{\alpha }+{C}_{1}^{\alpha }\left({S}_{0}+{I}_{0}+{V}_{0}+{R}_{0}\right)+{r}_{1}^{\alpha }}{1+\psi {\left(h\right)}^{\alpha }{\mu }^{\alpha }},$$

$${S}_{1}+{I}_{1}+{V}_{1}+{R}_{1}=\frac{\Lambda \psi {\left(h\right)}^{\alpha }+{C}_{1}^{\alpha }+{r}_{1}^{\alpha }}{1+\psi {\left(h\right)}^{\alpha }{\mu }^{\alpha }},$$

$${S}_{1}+{I}_{1}+{V}_{1}+{R}_{1}=\frac{\Lambda \psi {\left(h\right)}^{\alpha }+\alpha +\frac{1}{\Gamma (1-\alpha )}}{1+\psi {\left(h\right)}^{\alpha }{\mu }^{\alpha }}, {S}_{1}+{I}_{1}+{V}_{1}+{R}_{1}=N(1,\alpha ),$$

Now for $n=1$ and $\alpha \in (\mathrm{0,1})$,

$${S}_{2}+{I}_{2}+{V}_{2}+{R}_{2}=\frac{\Lambda \psi {\left(h\right)}^{\alpha }+\sum_{\nu =1}^{2}{C}_{\nu }^{\alpha }\left({S}_{2-\nu }+{I}_{2-\nu }+{V}_{2-\nu }+{R}_{2-\nu }\right)+{r}_{2}^{\alpha }}{1+\psi {\left(h\right)}^{\alpha }{\mu }^{\alpha }},$$

$${S}_{2}+{I}_{2}+{V}_{2}+{R}_{2}=\frac{\Lambda \psi {\left(h\right)}^{\alpha }+{{C}_{1}^{\alpha }\left({S}_{1}+{I}_{1}+{V}_{1}+{R}_{1}\right)+{C}_{2}^{\alpha }+r}_{2}^{\alpha }}{1+\psi {\left(h\right)}^{\alpha }{\mu }^{\alpha }},$$

$${S}_{2}+{I}_{2}+{V}_{2}+{R}_{2}<\frac{\Lambda \psi {\left(h\right)}^{\alpha }+{{C}_{1}^{\alpha }N(1,\alpha )+{C}_{2}^{\alpha }+r}_{1}^{\alpha }}{1+\psi {\left(h\right)}^{\alpha }{\mu }^{\alpha }} ,$$ from lemma 3, $${r}_{2}^{\alpha }<{r}_{1}^{\alpha },$$

$${S}_{2}+{I}_{2}+{V}_{2}+{R}_{2}<\frac{\Lambda \psi {\left(h\right)}^{\alpha }+N\left(1,\alpha \right)\left({C}_{1}^{\alpha }+{C}_{2}^{\alpha }\right)+\frac{1}{\Gamma (1-\alpha )}}{1+\psi {\left(h\right)}^{\alpha }{\mu }^{\alpha }} , N\left(1,\alpha \right)>1,$$

$${S}_{2}+{I}_{2}+{V}_{2}+{R}_{2}<\frac{\Lambda \psi {\left(h\right)}^{\alpha }+N\left(1,\alpha \right)\sum_{\nu =1}^{\infty }{C}_{\nu }^{\alpha }+\frac{1}{\Gamma (1-\alpha )}}{1+\psi {\left(h\right)}^{\alpha }{\mu }^{\alpha }},$$

$${S}_{2}+{I}_{2}+{V}_{2}+{R}_{2}<\frac{\Lambda \psi {\left(h\right)}^{\alpha }+N\left(1,\alpha \right)+\frac{1}{\Gamma (1-\alpha )}}{1+\psi {\left(h\right)}^{\alpha }{\mu }^{\alpha }} ,$$ from lemma 3, $$\sum_{\nu =1}^{\infty }{C}_{\nu }^{\alpha }=1,$$

$${S}_{2}+{I}_{2}+{V}_{2}+{R}_{2}<N(2,\alpha ).$$

where $$N\left(2,\alpha \right)=\frac{\Lambda \psi {\left(h\right)}^{\alpha }+N\left(1,\alpha \right)+\frac{1}{\Gamma (1-\alpha )}}{1+\psi {\left(h\right)}^{\alpha }{\mu }^{\alpha }}.$$

Similarly, for $n=0,$ we arrive at;

$${S}_{3}+{I}_{3}+{V}_{3}+{R}_{3}=\frac{\Lambda \psi {\left(h\right)}^{\alpha }+{{C}_{1}^{\alpha }\left({S}_{2}+{I}_{2}+{V}_{2}+{R}_{2}\right)+{C}_{2}^{\alpha }\left({S}_{1}+{I}_{1}+{V}_{1}+{R}_{1}\right)+{C}_{3}^{\alpha }+r}_{3}^{\alpha }}{1+\psi {\left(h\right)}^{\alpha }{\mu }^{\alpha }},$$

$${S}_{3}+{I}_{3}+{V}_{3}+{R}_{3}<\frac{\Lambda \psi {\left(h\right)}^{\alpha }+{{C}_{1}^{\alpha }N\left(2,\alpha \right)+{C}_{2}^{\alpha }N(1,\alpha )+{C}_{3}^{\alpha }+r}_{1}^{\alpha }}{1+\psi {\left(h\right)}^{\alpha }{\mu }^{\alpha }},$$

$${S}_{3}+{I}_{3}+{V}_{3}+{R}_{3}<\frac{\Lambda \psi {\left(h\right)}^{\alpha }+N\left(2,\alpha \right)\left({C}_{1}^{\alpha }+{C}_{2}^{\alpha }+{C}_{3}^{\alpha }\right)+\frac{1}{\Gamma (1-\alpha )}}{1+\psi {\left(h\right)}^{\alpha }{\mu }^{\alpha }},$$

$${S}_{3}+{I}_{3}+{V}_{3}+{R}_{3}<\frac{\Lambda \psi {\left(h\right)}^{\alpha }+N\left(2,\alpha \right)\sum_{\nu =1}^{\infty }{C}_{\nu }^{\alpha }+\frac{1}{\Gamma (1-\alpha )}}{1+\psi {\left(h\right)}^{\alpha }{\mu }^{\alpha }}=N(3,\alpha ),$$

$${S}_{3}+{I}_{3}+{V}_{3}+{R}_{3}<N(3,\alpha ).$$

Now, we suppose that for $n\in \left\{\mathrm{3,4},\dots ,{N}_{0}-1\right\}$ and for $\alpha \in \left(\mathrm{0,1}\right),$

$${S}_{{N}_{0}}+{I}_{{N}_{0}}+{V}_{{N}_{0}}+{R}_{{N}_{0}}<\frac{N\left({N}_{0}-1,\alpha \right)+\frac{1}{\Gamma (1-\alpha )}+\Lambda \psi {\left(h\right)}^{\alpha }}{1+\psi {\left(h\right)}^{\alpha }{\mu }^{\alpha }}=N\left({N}_{0},\alpha \right).$$

Thus, for $={N}_{0}$, we obtain:

$$\begin{aligned} & S_{n + 1} + I_{n + 1} + V_{n + 1} + R_{n + 1} \\ & = \frac{{{\Lambda }\psi \left( h \right)^{\alpha } + \mathop \sum \nolimits_{\nu = 1}^{n + 1} C_{\nu }^{\alpha } \left( {S_{n + 1 - \nu } + I_{n + 1 - \nu } + V_{n + 1 - \nu } + R_{n + 1 - \nu } } \right) + r_{n + 1}^{\alpha } }}{{1 + \psi \left( h \right)^{\alpha } \mu^{\alpha } }}, \\ \end{aligned}$$

$$\begin{aligned} & S_{{N_{0} + 1}} + I_{{N_{0} + 1}} + V_{{N_{0} + 1}} + R_{{N_{0} + 1}} \\ & = \frac{{{\Lambda }\psi \left( h \right)^{\alpha } + \mathop \sum \nolimits_{\nu = 1}^{{N_{0} + 1}} C_{\nu }^{\alpha } \left( {S_{{N_{0} + 1 - \nu }} + I_{{N_{0} + 1 - \nu }} + V_{{N_{0} + 1 - \nu }} + R_{{N_{0} + 1 - \nu }} } \right) + r_{{N_{0} + 1}}^{\alpha } }}{{1 + \psi \left( h \right)^{\alpha } \mu^{\alpha } }}, \\ \end{aligned}$$

$$\begin{aligned} & S_{{N_{0} + 1}} + I_{{N_{0} + 1}} + V_{{N_{0} + 1}} + R_{{N_{0} + 1}} \\ & = \frac{{{\Lambda }\psi \left( h \right)^{\alpha } + C_{1}^{\alpha } \left( {S_{{N_{0} }} + I_{{N_{0} }} + V_{{N_{0} }} + R_{{N_{0} }} } \right) + C_{2}^{\alpha } \left( {S_{{N_{0} - 1}} + I_{{N_{0} - 1}} + V_{{N_{0} - 1}} + R_{{N_{0} - 1}} } \right) + \ldots + C_{{N_{0} + 1}}^{\alpha } \left( {S_{0} + I_{0} + V_{0} + R_{0} } \right) + r_{{N_{0} + 1}}^{\alpha } }}{{1 + \psi \left( h \right)^{\alpha } \mu^{\alpha } }}, \\ \end{aligned}$$

$$\begin{aligned} & S_{{N_{0} + 1}} + I_{{N_{0} + 1}} + V_{{N_{0} + 1}} + R_{{N_{0} + 1}} \\ & < \frac{{{\Lambda }\psi \left( h \right)^{\alpha } + C_{1}^{\alpha } N\left( {N_{0} ,\alpha } \right) + C_{2}^{\alpha } N\left( {N_{0} - 1,\alpha } \right) + C_{3}^{\alpha } N\left( {N_{0} - 2,\alpha } \right) + \ldots + C_{{N_{0} }}^{\alpha } N\left( {1,\alpha } \right) + C_{{N_{0} + 1}}^{\alpha } + r_{1}^{\alpha } }}{{1 + \psi \left( h \right)^{\alpha } \mu^{\alpha } }}, \\ \end{aligned}$$

$$\begin{aligned} & S_{{N_{0} + 1}} + I_{{N_{0} + 1}} + V_{{N_{0} + 1}} + R_{{N_{0} + 1}} \\ & < \frac{{{\Lambda }\psi \left( h \right)^{\alpha } + C_{1}^{\alpha } N\left( {N_{0} ,\alpha } \right) + C_{2}^{\alpha } N\left( {N_{0} ,\alpha } \right) + C_{3}^{\alpha } N\left( {N_{0} ,\alpha } \right) + \ldots + C_{{N_{0} }}^{\alpha } N\left( {N_{0} ,\alpha } \right) + C_{{N_{0} + 1}}^{\alpha } N\left( {N_{0} ,\alpha } \right) + r_{1}^{\alpha } }}{{1 + \psi \left( h \right)^{\alpha } \mu^{\alpha } }}, \\ \end{aligned}$$

$${S}_{{N}_{0}+1}+{I}_{{N}_{0}+1}+{V}_{{N}_{0}+1}+{R}_{{N}_{0}+1}<\frac{\Lambda \psi {\left(h\right)}^{\alpha }+N({N}_{0},\alpha )\sum_{\nu =1}^{{N}_{0}+1}{C}_{\nu }^{\alpha }+{r}_{1}^{\alpha }}{1+\psi {\left(h\right)}^{\alpha }{\mu }^{\alpha }},$$

$${S}_{{N}_{0}+1}+{I}_{{N}_{0}+1}+{V}_{{N}_{0}+1}+{R}_{{N}_{0}+1}<\frac{N\left({N}_{0},\alpha \right)\sum_{\nu =1}^{\infty }{C}_{\nu }^{\alpha }+{r}_{1}^{\alpha }+\Lambda \psi {\left(h\right)}^{\alpha }}{1+\psi {\left(h\right)}^{\alpha }{\mu }^{\alpha }},$$

$${S}_{{N}_{0}+1}+{I}_{{N}_{0}+1}+{V}_{{N}_{0}+1}+{R}_{{N}_{0}+1}<\frac{N\left({N}_{0},\alpha \right)+\frac{1}{\Gamma (1-\alpha )}+\Lambda \psi {\left(h\right)}^{\alpha }}{1+\psi {\left(h\right)}^{\alpha }{\mu }^{\alpha }}=N\left({N}_{0}+1,\alpha \right).$$

Equivalently, it can be described as:

$${S}_{n+1}+{I}_{n+1}+{V}_{n+1}+{R}_{n+1}<N\left({N}_{0}+1,\alpha \right)$$ $$\forall n=\mathrm{1,2},3,\dots ,{N}_{0},$$ where $$0<\alpha <1.$$

Finally, ${S}_{n+1}, {I}_{n+1}, {I}_{n+1}, {R}_{n+1}\le N\left({N}_{0}+1,\alpha \right)$ for $=0, 1, 2, \dots ,{N}_{0}$ , as desired.

#### Remark

(1) $$N\left({N}_{0}+1,\alpha \right)>N\left({N}_{0},\alpha \right)>N\left({N}_{0}-1,\alpha \right)>\dots >N\left(1,\alpha \right)>1.$$

(2) $$N\left({N}_{0}+1,\alpha \right)\to 1 , as \alpha \to {1}^{-}.$$

## Discussion

In our current world, interdisciplinary research stands as a vital tool for ensuring the well-being and standard of living for all humanity. Over time, mathematical prediction modelling has become indispensable for understanding the behavior of epidemics, aiding policymakers in making crucial decisions and preparing for future challenges. Our study aimed to develop a mathematical model to predict the dynamics of COVID-19, employing the SIVR modelling framework. Scientists globally are striving to identify effective control measures to curb the spread of the COVID-19 virus. Strategies such as social distancing, quarantine for exposed individuals, isolation of infected persons, and mask-wearing are widely adopted. The primary objective in containing the virus is to minimize contact between susceptible and infected individuals, thereby reducing the transmission rate from the susceptible (S) to the infected (I) class.

Graphs plotted at the endemic equilibrium point illustrate the dynamical behavior of the susceptible population for varying fractional order parameter α, as depicted in Fig. 2. Each curve's trajectory demonstrates the susceptible compartment's convergence to the endemic steady state for different α values and specific parameter sets. Notably, the graphs display distinctive trajectories and convergence rates corresponding to α values, influencing the disease dynamics. Similarly, Fig. 3 exhibits curved graphs demonstrating the convergence of the infected compartment towards the fixed point, showcasing different phases of disease dynamics based on α values. Lower α values indicate slower convergence rates and vice versa, aligning with real-world system behaviors.

Figure 4 presents a set of graphs illustrating the critical role of vaccination in the COVID-19 model. These graphs depict the evolutionary behavior of vaccinated individuals during the disease propagation, with each graph exhibiting unique trajectories and convergence rates. The fractional order parameter α significantly influences the disease dynamics of each compartment.

Furthermore, Fig. 5 displays the dynamic behavior of the recovered population in persistent COVID-19 scenarios. The graphs highlight that a higher α value captures faster recovery rates, while lower α values represent slower recovery rates. Vaccination plays a pivotal role in disease control, particularly in managing COVID-19. Figure 5 illustrates the impact of vaccination on halting virus spread, with graphs drawn at α = 0.8 and varying vaccination parameter values δ_1_. Lower δ_1_ values result in a greater number of infected individuals compared to higher values of δ_1_ (e.g., δ_1_ = 0.2, 0.3, and 0.4). Thus, the number of infected individuals (I(t)) is inversely proportional to vaccination parameters δ_1_, underscoring its significant role in mitigating disease dynamics and effectively controlling virus transmission through widespread vaccination efforts.

## Concluding remarks

The fractional order COVID-19 model is developed to study the virus propagation in the population. To this end, analytical and numerical results are established. These results ascertained the unique positive solution of the underlying model. For numerical solutions, the GL-NSFD scheme is formulated to study the numerical aspects of the proposed scheme. This scheme has provided the positive and bounded numerical solutions, which are the salient features of the compartmental models. It is also identified that the disease model has two equilibrium state i.e. virus free and virus existing state. The simulated graphs and rendered that the endemic equilibrium may be obtained physically. Moreover, the disease-free state may also be attained in real scenario. Stability analysis is arranged and results for local and global stability are established for different values of ${R}_{0}$ i.e. ${R}_{0}<1$ or ${R}_{0}>1$. The results have shown that the fractional COVID-19 system preserves the stability at both the steady states depending upon the values of ${R}_{0}$. The crowding effect of the corona virus on the disease dynamics is presented. The role of vaccination for controlling the virus spread is also studied graphically. It is observed that the virus may be controlled, significantly if maximum portion of the population is vaccinated. Moreover, the SOP’s planned by the world health organization can reduce the rate of virus propagation in the society. Consequently, our proposed fractional epidemic model for the COVID-19 may be considered for studying the disease dynamics in the community. In addition, crowding effect and vaccination strategy can play a vital role devising the health policies and pre cautionary measures. Moreover, the fractional order epidemic model will be helpful for studying the dynamics of various disease in future.

In reading through the referee reports, we've noted that a number of additional specific references have been suggested. We would like to emphasize that we do not expect a research article to be comprehensive in its referencing of the background literature and that these additions would be at your discretion. Not including references which are not directly relevant to your work would not affect the final decision. However, please do ensure that directly relevant previous work is adequately acknowledged in your paper and that the original contribution of your study to furthering the current understanding in the field is sufficiently emphasized.

## Data Availability

The datasets used and analyzed during the current study available from the corresponding author on reasonable request.
